# Developability Assessment of Physicochemical Properties and Stability Profiles of HIV-1 BG505 SOSIP.664 and BG505 SOSIP.v4.1-GT1.1 gp140 Envelope Glycoprotein Trimers as Candidate Vaccine Antigens

**DOI:** 10.1016/j.xphs.2019.01.033

**Published:** 2019-07

**Authors:** Neal Whitaker, John M. Hickey, Kawaljit Kaur, Jian Xiong, Nishant Sawant, Albert Cupo, Wen-Hsin Lee, Gabriel Ozorowski, Max Medina-Ramírez, Andrew B. Ward, Rogier W. Sanders, John P. Moore, Sangeeta B. Joshi, David B. Volkin, Antu K. Dey

**Affiliations:** 1 Department of Pharmaceutical Chemistry, Macromolecule and Vaccine Stabilization Center, University of Kansas, Lawrence, Kansas 66047; 2 Department of Microbiology and Immunology, Weill Medical College of Cornell University, New York, New York 10065; 3 Department of Integrative Structural and Computational Biology, Center for HIV/AIDS Vaccine Immunology and Immunogen Discovery, International AIDS Vaccine Initiative Neutralizing Antibody Center, Collaboration for AIDS Vaccine Discovery, The Scripps Research Institute, La Jolla, California 92037; 4 Department of Medical Microbiology, Academic Medical Center, University of Amsterdam, Amsterdam, the Netherlands; 5 Product Development Center, International AIDS Vaccine Initiative, New York, New York 10004

**Keywords:** BG505, gp140, trimers, HIV-1 Env, developability assessment, stability, formulation, vaccine

## Abstract

The induction of broadly neutralizing antibodies (bNAbs) is a major goal in the development of an effective vaccine against HIV-1. A soluble, trimeric, germline (gI) bNAb-targeting variant of the HIV-1 envelope glycoprotein (termed BG505 SOSIP.v4.1-GT1.1 gp140, abbreviated to GT1.1) has recently been developed. Here, we have compared this new immunogen with the parental trimer from which it was derived, BG505 SOSIP.664 gp140. We used a comprehensive suite of biochemical and biophysical methods to determine physicochemical similarities and differences between the 2 trimers, and thereby assessed whether additional formulation development efforts were needed for the GT1.1 vaccine candidate. The overall higher order structure and oligomeric states of the 2 vaccine antigens were quite similar, as were their thermal, chemical, and colloidal stability profiles, as evaluated during accelerated stability studies. Overall, we conclude that the primary sequence changes made to create the gl bNAb-targeting GT1.1 trimer did not detrimentally affect its physicochemical properties or stability profiles from a pharmaceutical perspective. This developability assessment of the BG505 GT1.1 vaccine antigen supports using the formulation and storage conditions previously identified for the parental SOSIP.664 trimer and enables the development of GT1.1 for phase I clinical studies.

## Introduction

Envelope glycoproteins (Env) have been a focus of HIV-1 vaccine research for over 30 years.^1^ Because Env spikes are the only virus-encoded antigen exposed on the viral surface, they are the only relevant targets for broadly neutralizing antibodies (bNAbs). The Env spike is composed of a trimer of noncovalently linked gp120 and gp41 glycoprotein heterodimers. The soluble BG505 SOSIP.664 gp140 trimer adopts a native-like conformation and presents most of the known bNAb epitope clusters.^2^, ^3^ When tested as an immunogen in several animal models, this trimer elicits neutralizing antibodies (NAbs) to the neutralization-resistant (Tier-2) autologous BG505.T332N virus.^4^ A soluble, stable, variant trimer termed BG505 SOSIP.664 gp140 has been manufactured under cGMP conditions for evaluation in phase I clinical trials.^5^ To support the cGMP manufacture of this trimer, analytical characterization and formulation development experiments were performed to identify conditions that will not only ensure stability of the protein drug product during storage, but also its compatibility with adjuvants that will be used in clinical studies.

An effective HIV-1 Env vaccine will need to induce a more broadly active set of NAbs that could protect against multiple HIV-1 strains, which remains a highly challenging task. One plausible approach involves using an immunogen that targets the human germline (gl) precursors for bNAbs, followed by maturation of the initiated antibody lineage(s) by the use of one or more Env immunogens.^6^, ^7^, ^8^, ^9^, ^10^, ^11^ To explore this concept, a variant of the BG505 SOSIP.664 gp140 trimer was developed that, *in vitro*, can engage the gl-precursors of bNAbs that target the trimer apex and the CD4-binding site.^11^ The new trimer, BG505 SOSIP.v4.1-GT1.1 gp140 (abbreviated to GT1.1), was created from the parental SOSIP.664 trimer by introducing 18 amino acid substitutions and a 7-residue deletion into the V2 loop, resulting in the removal of 5 glycosylation sites.^11^ Despite these changes, the GT1.1 trimer retains the native-like conformation adopted by its parental molecule.^11^

As we plan to advance the GT1.1 trimer to early clinical development, we wanted to perform an early developability assessment to understand (1) formulation risks (and whether additional formulation development efforts are needed to produce a stable vaccine dosage form to facilitate rapid initiation of clinical evaluations), and (2) comparative physicochemical properties of GT1.1 trimer, in relation to the parental SOSIP.664 trimer, to evaluate the similarities and difference when formulated in the same (parental trimer) formulation condition. Thus, this work is a case study of evaluating the formulation/stability risk with a new complex protein-based HIV Env vaccine candidate by comparison to a closely related, yet molecularly distinct, HIV Env trimer antigen. Although developability assessments are commonly performed for selection of optimal molecular versions of a new monoclonal antibody drug candidate in terms of improved pharmaceutical properties (with similar biological properties^12^), this type of evaluation has not been as widely applied to new recombinant protein antigens during early vaccine development.

To this end, the biophysical and biochemical properties of both the SOSIP.664 and GT1.1 recombinant protein trimeric antigens have been systematically evaluated using a set of orthogonal physicochemical techniques that address key aspects of the structural integrity of both molecules. In addition, the conformational, chemical and colloidal stability profiles of both trimers were also evaluated and compared in the currently used formulation buffer for frozen liquid storage of the SOSIP.664 antigen. The prototypic BG505 SOSIP.664 trimer was first analyzed using a larger set of biophysical and biochemical methods. The resulting information allowed for down-selection of the most suitable procedures to apply to the GT1.1 trimer, which was only available in smaller quantities at this stage of the development process. Some of these methods have been used previously for analytical characterization and cGMP quality control analyses for SOSIP.664 gp140^13^, ^14^. Overall, we conclude that the primary sequence changes made to create the gl bNAb-targeting GT1.1 trimer did not detrimentally affect its physicochemical properties or stability profiles from a pharmaceutical perspective. Hence, the new GT1.1 vaccine antigen can be formulated and stored as frozen liquid dosage form under the conditions previously defined for the parental BG505 SOSIP.664 trimer,^5^ a finding that helps facilitate more rapid advancement of the new GT1.1 vaccine antigen into initial clinical development.

## Materials and Methods

### Materials

A frozen stock of BG505 SOSIP.664 gp140 trimers produced in stable CHO cells and purified by a series of column steps and formulated at a concentration of 3 mg/mL in “Formulation Buffer” (20 mM Tris, 100 mM NaCl pH 7.5)^5^ was supplied by KBI Biopharma Inc. The BG505 SOSIP.v4.1-GT1.1 trimer (GT1.1) is a modified version of BG505 SOSIP.664, engineered to engage germline antibody precursors, achieved by substitution of 18 amino acids (resulting in the removal of 5 potential N-glycan sites) and deletion of 7 amino acids in the V2 loop.^15^ The GT1.1 trimers were produced in stable CHO cells at the Weill Cornell Medical College, purified by the same method and supplied frozen at a concentration of 0.5 mg/mL in the same buffer. To use in method development (sedimentation velocity analytical ultracentrifugation [SV-AUC], size-exclusion chromatography [SEC], etc.), a 250-μg aliquot of CHO cell–expressed BG505 SOSIP.664 gp140 monomers (i.e., one protomer of the trimer) was collected from a 2G12-immunoaffinity and SEC column purification at the Weill Cornell Medical College. Sodium chloride was purchased from Fisher Scientific (Hampton, NH). Sucrose, trehalose from Pfanstiehl Laboratories (Waukegan, Illinois), and sulfobutyl-β-cyclodextrin from Captisol (San Diego, CA). All other chemicals were obtained from Sigma-Aldrich (St. Louis, MO).

### Methods

#### Sample Preparation

Before analysis, BG505 SOSIP.664 and GT1.1 gp140 trimer samples were thawed at room temperature and diluted in “Formulation Buffer” (see the aforementioned) to the desired concentration. Because the pH of Tris-buffered solutions is temperature-dependent, for some techniques described in the following (specifically, those that include a thermal melt), the trimers were dialyzed into PBS buffer (20 mM NaH_2_PO_4_, 100 mM NaCl pH 7.5) overnight at 4°C before use.

#### UV-Visible Spectroscopy

The UV-visible absorption spectra of the SOSIP.664 and GT1.1 trimers were recorded with an Agilent 8453 UV-visible spectrophotometer (Palo Alto, CA). The concentration of each protein was calculated based on the calculated^16^ extinction coefficients 1.590 g/L at 280 nm for SOSIP.664 and 1.559 g/L at 280 nm for GT1.1. Samples were assayed before and after centrifugation (5000 rpm for 5 min). Spectra were corrected for light scattering using a technique included in the manufacturer’s data analysis software (ChemStation UV-Vis analysis software, Agilent Technologies). Second derivative analysis was performed using Origin (OriginLab, Northampton, MA).

#### SDS-PAGE and BN-PAGE

BG505 SOSIP.664 and GT1.1 samples were mixed with 4X NuPAGE LDS sample buffer (Thermo Fisher Scientific) with and without 5 mM dithiothreitol (Thermo Fisher Scientific) and incubated at 95°C for 5 min. Samples were then treated with 10 mM iodoacetamide (Thermo Fisher Scientific) at 25°C in the dark for 30 min. Approximately, 10 μg of each sample were separated on 4%-12% Bis-Tris gels using NuPAGE MOPS SDS Running Buffer (Thermo Fisher Scientific). SeeBlue Plus2 Pre-Stained Protein Standard (Thermo Fisher Scientific) was used as a molecular weight (MW) ladder. BN-PAGE was performed with the same system used in SDS-PAGE. For Blue-Native gels, samples were mixed with NativePAGE Sample Buffer (Thermo Fisher Scientific) and were separated on NativePAGE Bis-Tris 3%-12% Mini Gels using NativeMark Unstained Protein Standard (Thermo Fisher Scientific) as a MW marker. Protein bands on all gels were visualized by staining with Bio-safe Coomassie Blue G250 stain (Bio-Rad Laboratories, Hercules, CA) or by silver staining.

#### LC-MS Peptide Mapping

Samples were prepared by drying 80 μg of protein using a SpeedVac (Eppendorf, Hamburg, Germany), followed by resuspension with 250 mM Tris, 6M guanidine HCl, pH 7.5, and reduction with 50 mg/mL DTT (55°C for 20 min). The samples were then alkylated with 50 mM iodoacetamide for 30 min at room temperature in the dark, before addition of 180 μL of 50 mM Tris pH 7.5 and 2 μL of PNGase F (Promega, Madison, WI) and incubation of the mixture overnight at 37°C. The following morning, either a trypsin/LysC mixture (Promega) or chymotrypsin (Promega) was added, and the samples were incubated for 4 h at 37°C. Trifluoroacetic acid (TFA, 0.5%) was added to quench the proteolysis reaction, and 50 μL of the digested protein solution was subjected to liquid chromatography–mass spectrometry (LC-MS), as follows.

The peptides from the digested protein solution were separated by a liquid chromatography system (Thermo Fisher Scientific) before analysis. Peptides were injected onto a C18 column (1.7 μm, 2.1 × 150 mm, Waters Corporation, Milford, MA) and a 90 min 0.5%-41% B gradient (A: H_2_O and 0.05% TFA; B: acetonitrile and 0.04% TFA; 200 μL/min flow rate) was used for separation. Mass spectrometry (MS) was performed using an LTQ-XL ion trap (Thermo Fisher Scientific) and the Xcalibur 2.0 software (Thermo Fisher Scientific). The instrument was also tuned using a standard calibration peptide (Angiotensin II, Sigma-Aldrich, St. Louis, MO) for maximal sensitivity before running any analyses. The mass spectra were acquired in the LTQ over a mass range of m/z 400-2000, the ion selection threshold was 10,000 counts and the dynamic exclusion duration was 8 s.

Raw experimental files were initially evaluated manually to determine if the ion counts and fragmentation of each peptide were sufficient for further analysis. The raw data files were then processed using PepFinder 2.0 software (Thermo Fisher Scientific). The database used for this experiment consisted of the BG505 SOSIP.664 or GT1.1 primary sequence. Potential Cys carbamidomethylation, Asn deamidation, and methionine (Met)/Trp/His/Cys oxidation events were considered during the analysis. Peptide assignments of MS/MS spectra were validated using a confidence score of ≥95%.

#### Size Exclusion, Cation Exchange, and Reversed-Phase High-Performance Liquid Chromatography

Before all HPLC experiments, all samples were centrifuged for 5 min at 12,000 × *g* to remove any large aggregates/particles that could interfere with the analysis. Size exclusion high-performance liquid chromatography was performed in triplicate with a Shimadzu HPLC system with UV absorbance detection at 220 with a Tosoh TSKgel Ultra SW Aggregate Column and a corresponding guard column. Experiments were performed at 25°C with a mobile phase containing 0.2 M sodium phosphate, 400 mM NaCl, and pH 7.5, and a flow rate of 0.5 mL/min was used to separate species based on size.

Cation exchange chromatography (CEX) was also performed on a Prominence UFLC system. Thirty microgarm of BG505 SOSIP.664 or GT1.1 trimers in formulation buffer were injected onto a TSKgel BioAssist S column (4.6mm ID × 5cm, PEEK column, Tosoh Biosciences). The flow rate was set at 0.5 mL/min and the column oven and autosampler temperatures were set at 30°C and 4°C, respectively. Elution was monitored using the absorbance at 214 and 280 nm. Mobile phase A consisted of 10 mM NaH_2_PO_4_, pH 7.0, whereas mobile phase B consisted of 10 mM NaH_2_PO_4,_ 1 M NaCl, pH 7.0. The mobile phase gradient consisted of 0% B (5 min), 0%-100% B (30 min), 100% B (3 min), and 0% B (2 min).

Reversed-phase high-performance liquid chromatography (RP-HPLC) experiments were performed on a Thermo UltiMate 3000 UHPLC system (Thermo Fisher Scientific) equipped with a diode array detector. Thirty microgram of BG505 SOSIP.664 or GT1.1 trimers in formulation buffer were injected onto a Waters ACQUITY UPLC Protein BEH C4 column (2.1 × 150 mm, 1.7 μm, Waters Corporation, Milford, MA). The flow rate was set at 0.2 mL/min and the column oven and autosampler temperatures were set at 25°C and 4°C, respectively. Elution was monitored using the absorbance at 214 nm. Mobile phase A consisted of H_2_O with 0.1% TFA and mobile phase B consisted of 0.1% TFA in 90:10 acetonitrile: H_2_O (v/v). The mobile phase gradient consisted of 35% B (5 min), 35%-65% B (30 min), 35%-95% B (4 min), 95% B (3 min), and 35% B (8 min).

#### Fourier-Transform Infrared Spectroscopy

Fourier-transform infrared spectroscopy (FTIR) spectroscopy was performed using a Bruker Tensor-27 FTIR spectrometer (Bruker Optics, Billerica, MA) equipped with a KBr beam splitter. The mercuric cadmium telluride detector was cooled with liquid N_2_ and the interferometer was constantly purged with N_2_ gas. All instrument validation tests were performed and passed before daily measurements. Two hundred and fifty six scans were recorded from 4000 to 600 cm^−1^ with a 4 cm^−1^ resolution using a Bio-ATR cell. Background measurements were acquired with formulation buffer alone and subtracted from the sample spectra. Atmospheric corrections, baseline corrections, and second derivative calculations were applied using OPUS V6.5 (Bruker Optics, Billerica, MA) software. Following Fourier self-deconvolution, between 6 and 8 peaks were fitted to the absorbance spectrum in the Amide I region (1700-1600 cm^−1^) using a mixed Gaussian and Lorentzian function. The areas of the peaks were used to determine the relative percentage of secondary structure components in the trimer samples.

#### Differential Scanning Calorimetry

Differential scanning calorimetry (DSC) was performed on the trimer samples in triplicate using an Auto-VP capillary differential scanning calorimeter (MicroCal/GE Health Sciences, Pittsburgh, PA) equipped with tantalum sample and reference cells. The SOSIP.664 or GT1.1 trimers (at 0.2 mg/mL), or PBS alone, were loaded in a DSC autosampler tray held at 4°C. Scans were completed from 10°C to 100°C using a scanning rate of 60°C/h. Reference subtraction and concentration normalization were performed using the instrument software. T_m_ and T_onset_ values were determined using Origin (OriginLab, Northampton, MA).

#### Fluorescence Spectroscopy

Intrinsic tryptophan fluorescence data for the SOSIP.664 and GT1.1 gp140 trimers were collected on a Fluorescence Innovations Plate Reader (Fluorescence Innovations Inc., Minneapolis, MN). Ten millimeter aliquots of 0.2 mg/mL samples were analyzed in sextuplicate in black 386-well plates. An excitation wavelength of 295 nm was used with a 315 nm filter. Samples were heated from 10°C to 90°C using a stepsize of 2.5°C. Peak position was calculated using Fluorescence Innovations data analysis software.

Eight-Anilino-1-naphthalene sulfonate (ANS) was used as an extrinsic fluorescence probe in the presence of the SOSIP.664 or GT1.1 trimers. Experiments were performed in PBS using an Agilent Stratagene Mx3005P QPCR System. A solution containing 100 μM ANS (generally used at 20× the molar concentration of protein) was excited at 372 nm, and the ANS emission spectrum was collected from 400 to 600 nm every 2 nm. The final protein concentration used was 0.2 mg/mL.

#### Data Visualization Using Three-Index Empirical Phase Diagrams

Three-index empirical phase diagrams (EPDs) were constructed as described previously^17^, ^18^ to analyze the large biophysical stability datasets generated as function of pH and temperature. Intrinsic Trp fluorescence peak position, ANS fluorescence peak intensity, and DSC data were used for construction of the 3-index EPDs for both gp140 proteins. All calculations were performed using the in-house software. Each temperature and pH pair was grouped in regions using a k-means clustering algorithm. The final definition of structural regions was confirmed by visual assessment of the EPD regions versus the trends observed in the biophysical data sets.

#### Dynamic Light Scattering

The dynamic light scattering (DLS) mode on a ZetaPALS zetasizer (Brookhaven Instruments Corporation, Holtsville, NY) was used, with quartz cuvettes that had been cleaned of any dust and air-dried. The hydrodynamic diameters of filtered SOSIP.664 or GT1.1 samples (at 0.5 mg/mL) were analyzed by generating an autocorrelation decay function after centrifugation at 14,000 × *g* for 5 min. Ten measurements were recorded and averaged for 30 s each. Number and intensity distributions were fitted using the cumulant analysis algorithm provided with the instrument software. All measurements were performed in triplicate at 25°C, and the viscosity of the solvent was 0.89 cP.

#### Sedimentation Velocity Analytical Ultracentrifugation

SV-AUC studies were conducted on a ProteomeLab XL-I analytical ultracentrifuge equipped with a scanning ultraviolet-visible optical system (Beckman Coulter, Fullerton, CA). All experiments were performed at 20°C with a rotor speed of 40,000 rpm and detection at 280 nm. Samples (0.5 mg/mL of SOSIP.664 or GT1.1) and formulation buffer alone were loaded into Beckman charcoal-epon 2 sector cells with a 12 mm centerpiece and quartz windows. Sednterp (Professor Thomas Laue, University of New Hampshire) was used to calculate the partial specific volume based on the primary sequence of the protein (around 0.73 mL/g) and the density and viscosity of the formulation buffer. Data were analyzed using Sedfit (Peter Schuck, NIH). A continuous c(s) distribution fitting model was applied with 50 scans. Frictional ratio, radial independent noise, and time independent noise were also fit, whereas the meniscus and bottom positions were set manually. A range of 0 to 25 Svedbergs was used, after verifying that there was no signal that sedimented outside of this range. A resolution of 300 points per distribution and a confidence level of 0.95 were used.

#### Microflow Imaging

Subvisible particle concentrations were assayed by microflow imaging (MFI) using an MFI 5200 instrument (ProteinSimple, Santa Clara, CA) equipped with a Bot1 autosampler. Before analysis, the instrument was calibrated using 10 μm polystyrene particle standards (Thermo Fisher Scientific), and it was cleaned with filtered water and 2% PCC-54 Detergent. BG505 SOSIP.664 samples were measured undiluted (at 3 mg/mL) while GT1.1 trimers were diluted 10-fold into formulation buffer (final concentration of 0.05 mg/mL) to conserve sample.

#### Biolayer Interferometry

BG505 SOSIP.664 and GT1.1 potencies were quantified by biolayer interferometry (BLI) using an Octet RED96 Bio-Layer Interferometry System (ForteBio, Menlo Park, CA). Binding experiments were performed with Protein A Biosensors (ForteBio) in 96-well half-area black plates. The biosensors were loaded with PGT145 antibodies at a final concentration of 1 μg/mL. Binding buffer (1× PBS, 0.1% polysorbate 20%, 0.1% BSA, pH 7.2) was used for all the baseline and disassociation wells as well as for diluting PGT145, SOSIP.664 or GT1.1. BG505 SOSIP.664 solutions at 300,150, 75, 37.5, 18.8, and 9.4 mg/mL were used in triplicate as standards. Samples were all analyzed at 100 mg/mL. Binding was performed for 300 s at 1000 rpm. Binding rates were calculated using the Octet Analysis Software (ForteBio).

#### Negative Staining Electron Microscopy

Trimer samples were diluted to ∼0.01-0.03 mg/mL in formulation buffer (see Sample Preparation) before negative staining electron microscopy (NS-EM) analysis. Previously described procedures were followed for grid preparation, imaging, and data analysis.^16^, ^17^ A 2% (w/v) solution of uranyl formate was used as the stain, and images were collected on an FEI Tecnai T12 electron microscope equipped with a Tietz TemCam-F416 CMOS camera (120 keV, 2.05 Å/pixel, ∼25 e^-^/Å^2^ total dose, 1500 nm nominal defocus).

## Results

### Comparative Physicochemical Characterization of BG505 SOSIP.664 and BG505 SOSIP.v4.1-GT1.1 gp140 Trimers

The primary structures of the 2 gp140 molecules (Supplemental Fig. S1) were confirmed and compared by performing LC-MS peptide mapping on BG505 SOSIP.664 (Supplemental Fig. S2) and GT1.1 gp140 proteins (Supplemental Fig. S3). The proteins were digested using either trypsin and LysC (Supplemental Figs. S2A and S3A) or chymotrypsin (Supplemental Figs. S2B and S3B). Chymotrypsin digestion resulted in better sequence coverage for SOSIP.664 gp140 (∼95%) than trypsin and LysC digestion (∼87%). Similarly, sequence coverage for GT1.1 was notably superior after chymotrypsin (94% coverage) digestion, compared to trypsin and LysC (80% coverage). Before analysis by LC-MS, the peptides were deglycosylated with PNGase to remove the N-linked glycans. The oligosaccharide content differences between the 2 trimers were not examined here because the glycan composition is not expected to change during storage. This LC-MS method can be used to assess whether chemical modifications of amino acid residues in the protein antigens (e.g., Asn deamidation, Met oxidation) occur during accelerated stability studies (see in the following).

**Figure 1:**
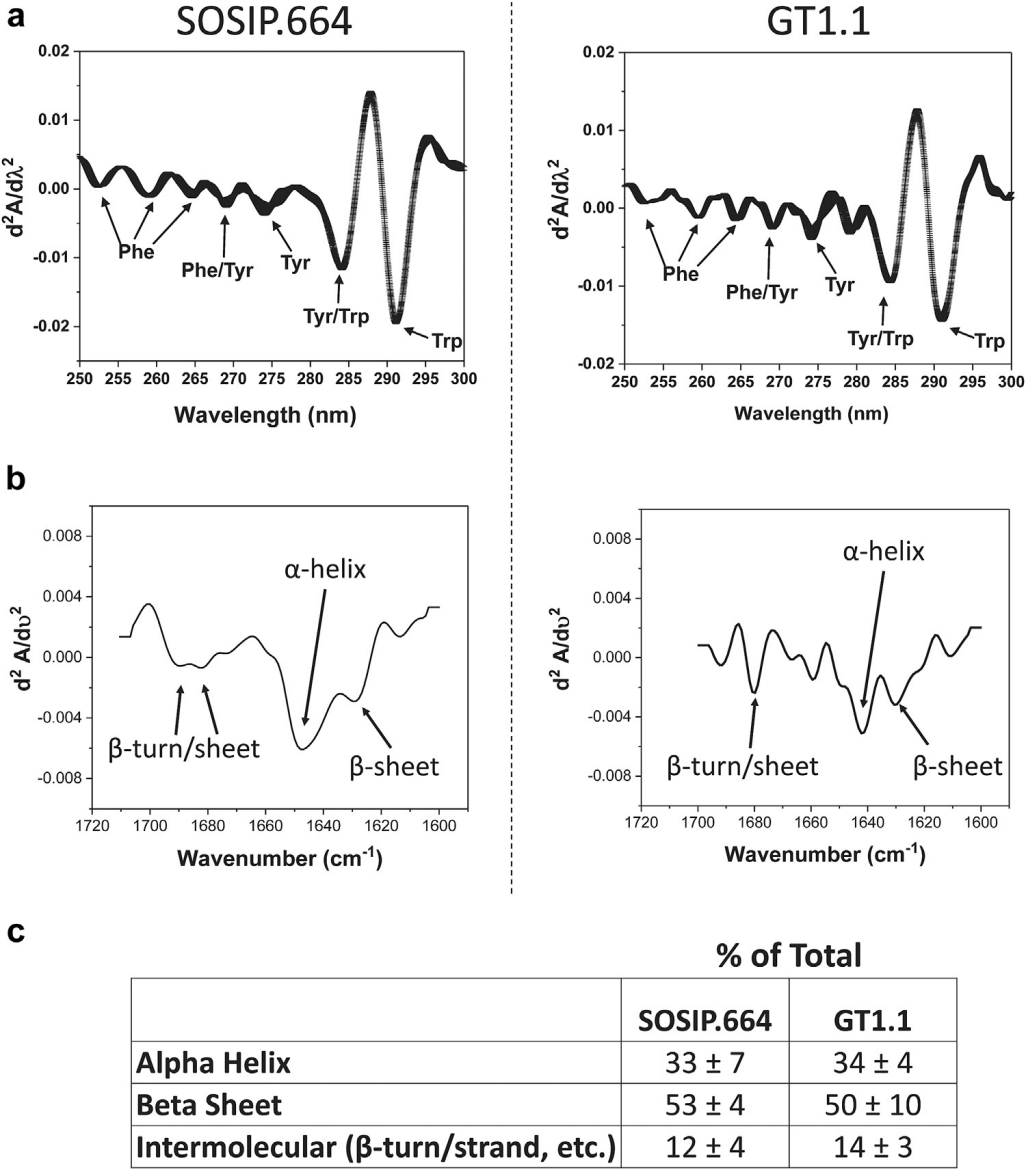
Overall secondary and tertiary structural analysis of BG505 SOSIP.664 (left) and GT1.1 (right) gp140 trimers. (a) Second-derivative UV-visible spectroscopy analysis. Error bars represent one standard deviation from triplicate spectra. (b) Representative second-derivative analyses of FTIR spectra. (c) Summary table of secondary structure composition of SOSIP.664 and GT1.1 as determined by FTIR. Error values represent standard deviation from triplicate measurements. See Supplemental Figure S4 for secondary structure determination by fitting Amide I region of IR spectra to 8 peaks using a mixed Gaussian and Lorentzian function.

The overall secondary and tertiary structures of the SOSIP.664 and GT1.1 gp140 trimers in solution were evaluated by FTIR and second-derivative UV-visible spectroscopy (Fig. 1), respectively. Small differences were observed between the second-derivative UV-visible spectra of the 2 gp140 trimers, indicative of subtle influences of the microenvironments of their aromatic amino acids, which likely arises from primary structure variation (Fig. 1a). FTIR analysis revealed that both proteins had similar overall secondary structure contents, comprising a mixture of α-helices and β-sheets, consistent with previous structural studies^19^ (Figs. 1b, 1c and Supplemental Fig. S4).

**Figure 2:**
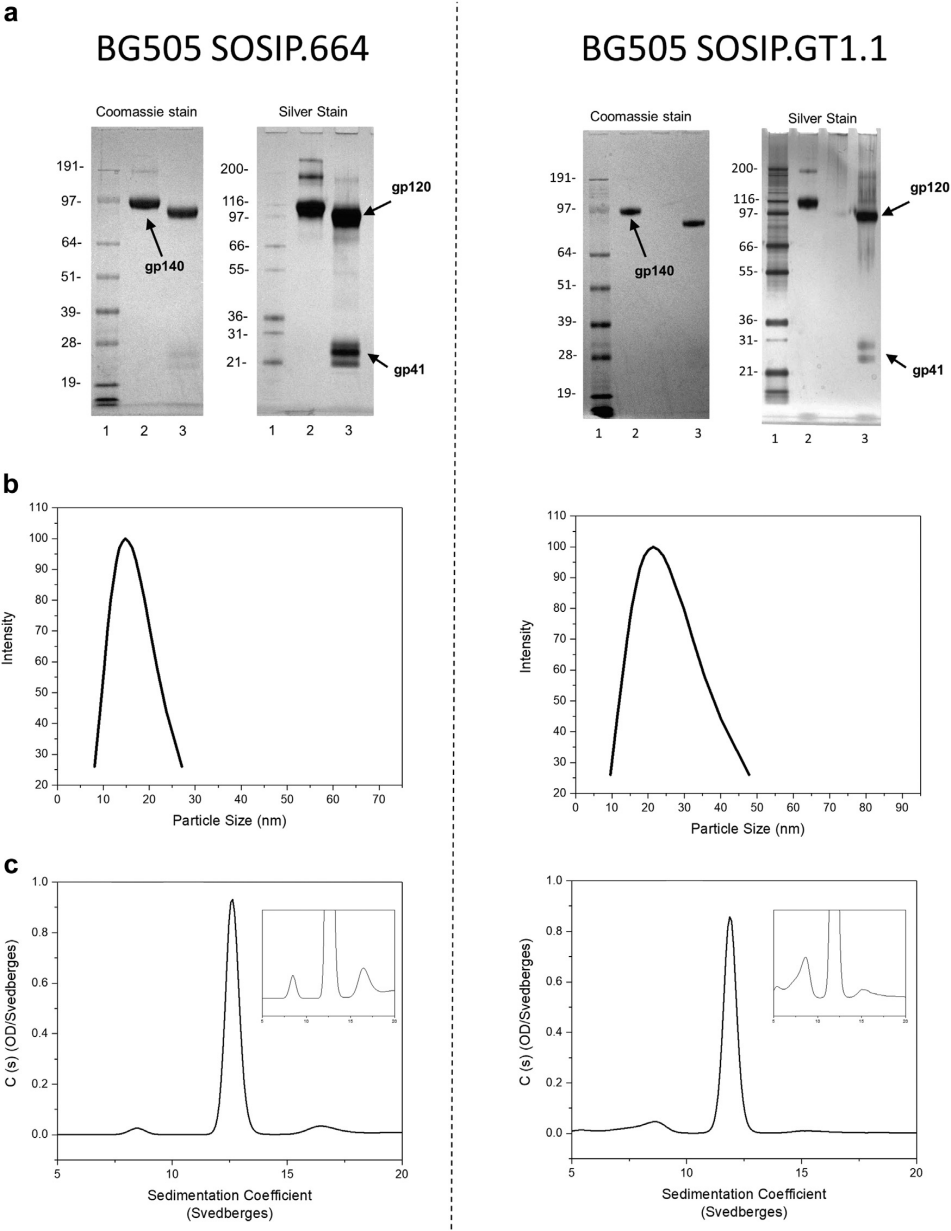
Molecular size analysis of BG505 SOSIP.664 (left) and GT1.1 (right) trimers under denaturing and nondenaturing conditions. (a) SDS-PAGE analysis using Coomassie blue or silver stain. Lane 1 contains molecular weight markers, lane 2 contains nonreduced BG505 protein and lane 2 contains reduced BG505 protein. In case of nonreduced, the predominant gp140 band is visible, whereas on reduction, gp120 and gp41 (ectodomain) are visible. (b) Dynamic light scattering data showing intensity-weighted cumulant (*y*-axis) analysis for SOSIP.664 (left) and GT1.1 (right) gp140 proteins. Particle sizes (in nm) are shown along *x*-axis. (c) Representative SV-AUC analysis of SOSIP.664 (left) and GT1.1 (right) with *y*-axis zoom shown in inset.

Size-based analysis of the SOSIP.664 and GT1.1 trimers was performed using a combination of denaturing (SDS-PAGE) and nondenaturing conditions (DLS and SV-AUC), as shown in Figure 2. The results of the SDS-PAGE analysis were similar to those reported previously,^2^ with nonreduced SOSIP.664 gp140 migrating as a single band and the reduced sample migrating as a single gp120 band and a poorly resolved gp41 ectodomain (gp41_ecto_) triplet (Fig. 2a, left panel). Similar results were observed for GT1.1 gp140 (Fig. 2a, right panel). The hydrodynamic diameters of the SOSIP.664 and GT1.1 gp140 proteins in solution were determined by DLS. Intensity-weighted cumulant analyses revealed diameters of 18.6 ± 1.6 nm and 22.9 ± 2.5 nm for SOSIP.664 and GT1.1 gp140 proteins, respectively (Fig. 2b). One explanation for this minor discrepancy would be the presence of small amounts of higher MW aggregates in the GT1.1 sample. This was confirmed to be the case when the DLS data were evaluated by an intensity-weighted mean square displacement analysis (Supplemental Fig. S5). In addition, a greater number of subvisible particles are present in GT1.1 samples when analyzed by MFI (see in the following). SV-AUC was then used as an orthogonal technique to estimate the MWs (and percent trimer) for SOSIP.664 and GT1.1. The SV-AUC profiles were similar for both trimers (Fig. 2c). The GT1.1 trimer population gave a calculated mass of 358 ± 8 kDa with a sedimentation coefficient value of 11.9 ± 0 S, whereas the corresponding values for SOSIP.664 were 373 ± 15 kDa and 12.3 ± 0 S (Supplemental Table S1). The differences in sedimentation coefficient values, and the estimated mass reduction of ∼14 kDa for GT1.1, are consistent with the deletion of 7 amino acid residues and 5 potential N-glycan sites when generating the GT1.1 trimer from the SOSIP.664 prototype (assuming an average amino acid MW of 110 Da and 2000 Da per glycan site). Size exclusion chromatography was also performed on SOSIP.664 (Supplemental Fig. S6) but was found to not be as informative as SV-AUC and was not used in analysis of GT1.1.

**Figure 3:**
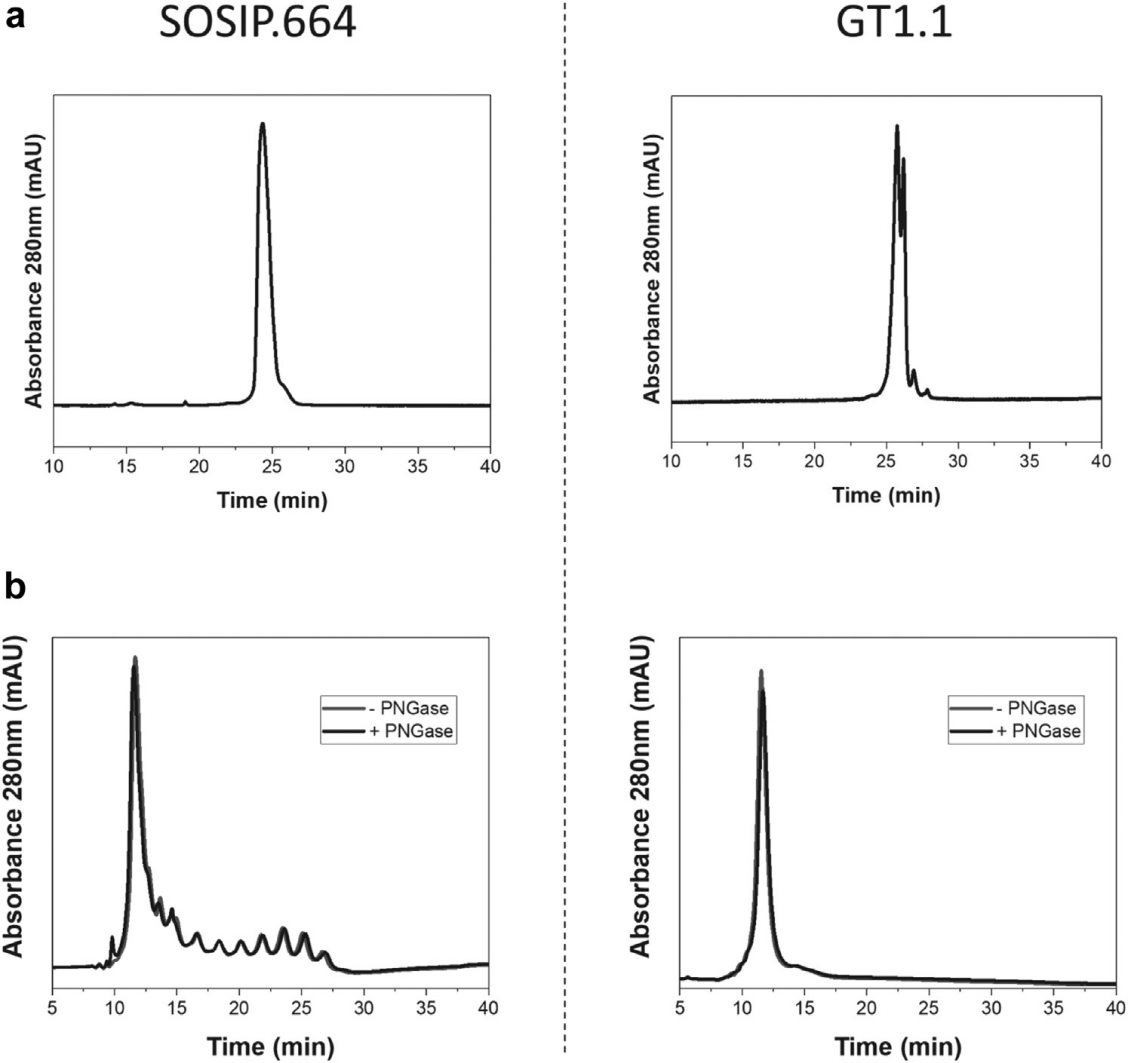
Charge heterogeneity and hydrophobicity comparison of BG505 SOSIP.664 (left) and GT1.1 (right) gp140 proteins by different chromatographic methods. (a) Representative RP-HPLC analyses of SOSIP.664 and GT1.1 gp140s. (b) Representative CEX-HPLC analyses of intact SOSIP.664 and GT1.1 gp140s (blue chromatogram) and PNGase F-treated deglycosylated SOSIP.664 and GT1.1 gp140s (black chromatogram).

Reversed-phase and cation exchange high-performance liquid chromatography (RP-HPLC, CEX-HPLC) were used to assess hydrophobic and charge heterogeneity, respectively, for the SOSIP.664 and GT1.1 gp140s (Fig. 3). In RP-HPLC, SOSIP.664 gp140 eluted as a single main peak comprising over 90% of the total area, but with 2 shoulders (Fig. 3a, left panel). Three minor peaks eluted earlier than the main peak but collectively comprised less than 1% of the total area of the chromatogram. The GT1.1 RP-HPLC chromatograms were notably different from that of SOSIP.664, in that the main species eluted as 2 overlapping peaks (Fig. 3a, right panel). Charge heterogeneity was evaluated by CEX-HPLC. Here, SOSIP.664 gp140 had a more heterogeneous profile than GT1.1 (Fig. 3b). Nearly all (>99%) of the loaded SOSIP.664 protein eluted from the column (determined by injecting sample with and without the column in line). One main peak (at ∼12.5 min) comprised ∼50% of the total peak area of the chromatograms but at least 10 individual later-eluting peaks were also identified (Fig. 3b, left panel). The GT1.1 profile in the CEX-HPLC analysis was markedly different; while the main peak eluted at approximately the same retention time as SOSIP.664, no later eluting peaks were observed (Fig. 3b, right panel). In an attempt to explain the apparent heterogeneity in the SOSIP.664 CEX chromatographic profile, we enzymatically digested the protein with PNGase F to remove the N-glycans from the protein (Fig. 3b and Supplemental Fig. S7). Although the SDS-PAGE data indicated that approximately ∼40 kDa of N-glycans were removed from each SOSIP.664 monomer (Supplemental Fig. S7), no observable changes were seen in the CEX chromatograms (Fig. 3b, left panel). Similarly, no change was seen in the SDS-PAGE and CEX analyses of PNGase F treated GT1.1 gp140 protein (Fig. 3b, right panel and Supplemental Fig. S7).

The aforementioned analyses imply that the GT1.1 trimer is more heterogeneous than SOSIP.664 from the perspective of hydrophobicity (RP-HPLC), but the converse applies to surface charge (CEX-HLPC) with SOSIP.664 being the more heterogeneous protein. The differences between the 2 proteins could be rooted in the amino acid sequence changes used to create the GT1.1 gp140 trimer from SOSIP.664, including variations in post-translational modifications such as how N-glycosylation sites are utilized. In addition, some of these differences may be due to differences in Met oxidation within each of the proteins (see below in the following results).

### Biophysical Stability Profile of BG505 SOSIP.664 and GT1.1 Trimers as a Function of Temperature and pH

**Figure 4:**
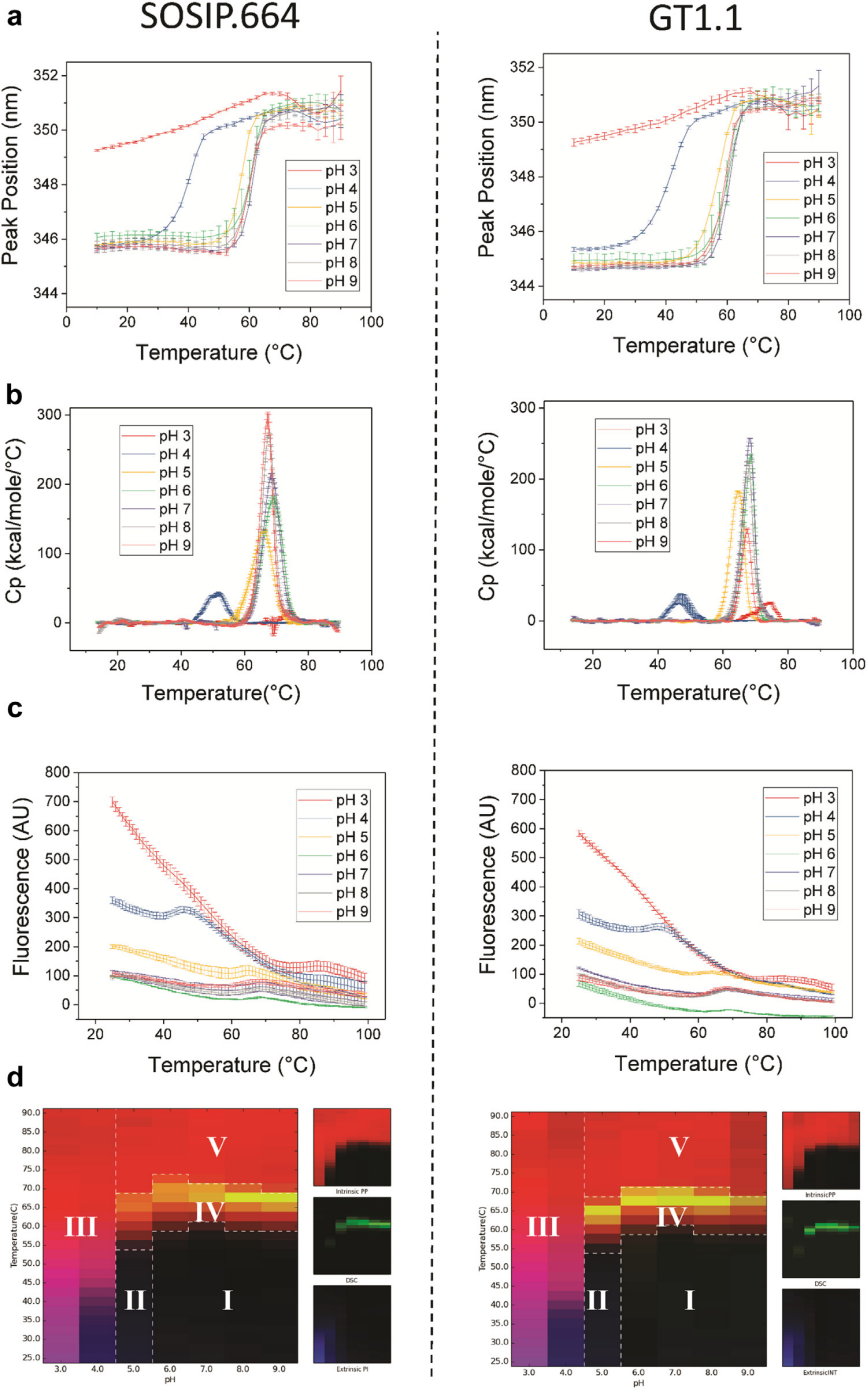
Conformational stability of BG505 SOSIP.664 (left) and GT1.1 (right) gp140 proteins as a function of pH and temperature. (a) Tryptophan fluorescence spectroscopy peak position in SOSIP.664 and GT1.1 as a function of temperature and pH. (b) Overall structural integrity of BG505.664 and GT1.1 as a function of temperature and pH as analyzed by DSC. (c) ANS fluorescence intensity as a function temperature and pH. Error bars represent standard deviation from triplicate measurements. (d) Data visualization using 3-index EPD analyses of biophysical stability data sets (a-c) for SOSIP.664 and GT1.1. See text for explanation of color scheme.

The conformational stability of SOSIP.664 gp140 trimers was evaluated across a pH range of 3.0 to 9.0 and varying temperatures by DSC, intrinsic tryptophan fluorescence peak position, and ANS fluorescence peak intensity (Figs. 4a-4c, respectively). All 3 methods indicated that lower pH values destabilized the SOSIP.664 trimers in a temperature-dependent manner; the overall tertiary structure of the protein molecules was affected at low pH, even at low temperatures, consistent with data reported earlier.^5^ At higher pH values (≥pH 6), thermal transitions were observed (Figs. 4a-4c) with calculated T_m_ values between 55°C and 70°C (Supplemental Fig. S8). These results are consistent with previous DSC studies.^2^, ^5^ Using these biophysical stability data sets, a 3-index EPD^17^, ^18^ was constructed to better visualize and compare the temperature and pH stability profiles of both molecules and to identify the contributions of each of the 3 individual techniques (Fig. 4d, left panel). Intrinsic tryptophan fluorescence peak position (red), extrinsic ANS fluorescence intensity (blue), and DSC (green) were used for this analysis, in which 5 distinct colored phases (designated as regions I-V) were identified. Specifically, in addition to the SOSIP.664 native state (region I), 4 structurally altered protein states (regions II-V) were observed. Regions II and III correspond to states that are structurally altered, to varying extents, under low pH conditions. Region IV represents an overall structurally altered conformation, reflecting the pH- and temperature-dependent unfolding processes observed by DSC and intrinsic fluorescence spectroscopy. Region V represents a more extensively structurally altered protein.

For comparison, the GT1.1 gp140 protein was evaluated using the same biophysical methods and by EPD analysis. Overall, its thermal and pH stability profiles were highly similar to SOSIP.664, with region I comprising 30% of the total EPD area for both molecules. The conformational stability profiles of the 2 molecules as a function of pH and temperature, again as measured by intrinsic tryptophan fluorescence, ANS fluorescence, and DSC, were also similar, and no notable differences in the thermal melting temperature values were identified (Figure 4, Figure 6 and Supplemental Fig. S8). As with SOSIP.664, 5 differently colored structural states were identified in the EPD for GT1.1 (Fig. 4d, right panel).

### Chemical Stability Profiles of BG505 SOSIP.664 and GT1.1: Oxidative Stress Studies

**Figure 5:**
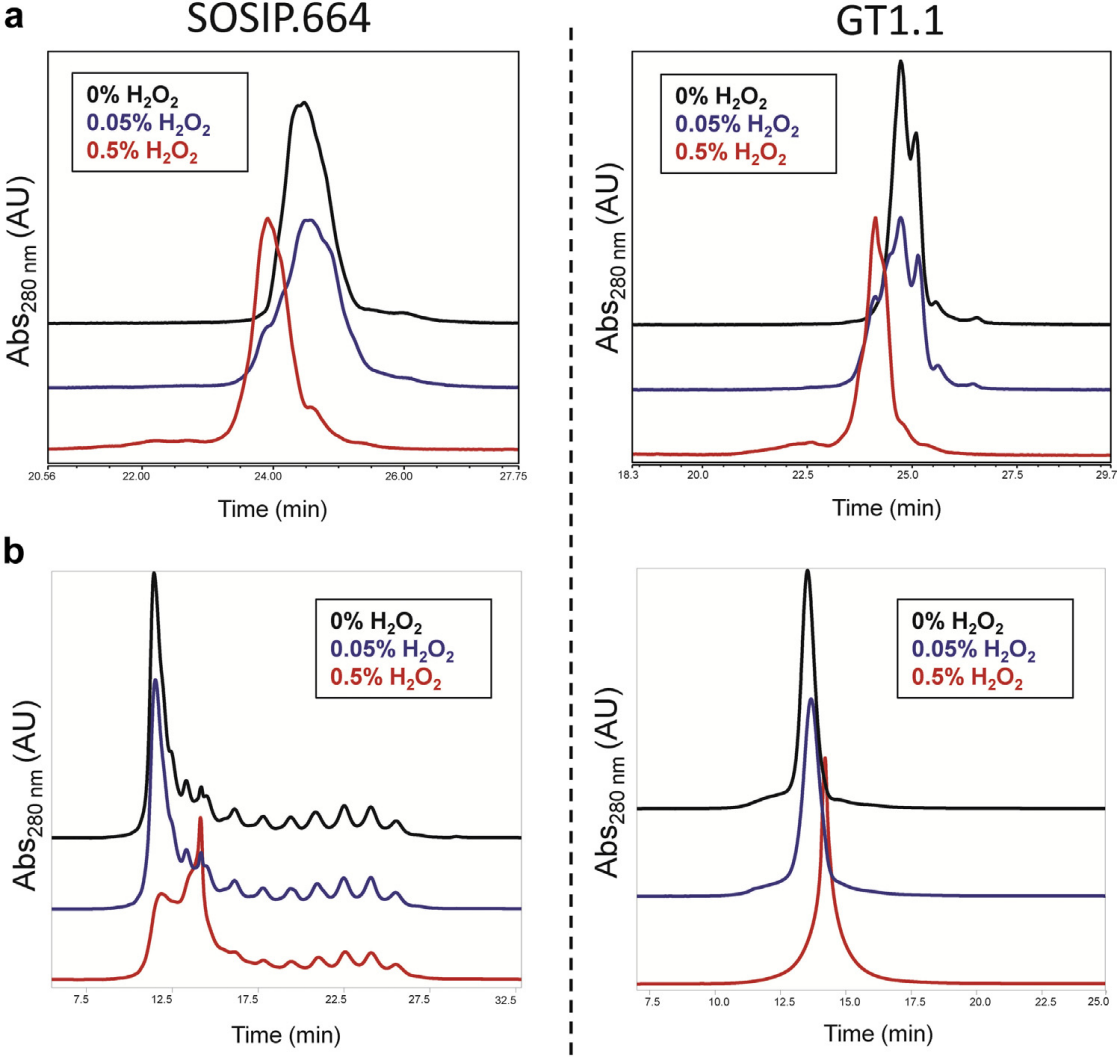
Chemical stability profile during forced oxidation studies of BG505 SOSIP.664 and GT1.1 gp140 trimers. (a) Representative RP-HPLC analyses of (0.05% and 0.5%) H_2_O_2_ stressed SOSIP.664 and GT1.1 samples in comparison to untreated control (0% H_2_O_2_). (b) Representative CEX-HPLC analyses of (0.05% and 0.5%) H_2_O_2_ stressed SOSIP.664 and GT1.1 samples in comparison to untreated control (0% H_2_O_2_).

Chemical stability profiles were determined by subjecting the SOSIP.664 and GT1.1 gp140 proteins to forced oxidation, followed by RP-HPLC, CEX-HPLC analyses, and LC-MS peptide mapping. The protein samples were exposed to 0%, 0.05%, and 0.5% H_2_O_2_ for 1 h at room temperature (Fig. 5). As described previously in Figure 3, the RP-HPLC and CEX-HPLC elution profiles of the nonstressed SOSIP.664 and GT1.1 samples (i.e., 0% H_2_O_2_) were notably different, which was also seen in this set of experiments. Thus, in the RP-HPLC chromatograms, GT1.1 eluted as 2 overlapping peaks (at ∼24.5 and ∼25.0 min), whereas only 1 peak (∼24.5 min) was seen for SOSIP.664 (Fig. 3a). In the presence of 0.05% H_2_0_2_, however, 2 closely eluting peaks were observed with SOSIP.664 (Fig. 5a). It is therefore possible that the single peak in the unstressed SOSIP.664 sample is composed of 2 coeluting species that behave differently on exposure to H_2_O_2_. We also saw that, in the presence of increasing concentrations of H_2_O_2_, the polarity of both gp140 trimer samples increased, resulting in earlier elution times. In both cases, the extent of this change in retention time was similar at the same H_2_O_2_ concentrations. The H_2_O_2_-induced changes to the elution profiles of the SOSIP.664 and GT1.1 gp140 trimers were also similar in the CEX-HPLC analysis (Fig. 5b), despite the notable differences between their elution profiles under nonstressed (control) conditions (Fig. 3b). Thus, in the presence of H_2_O_2_, for both samples, the area of the main peak began to decrease and shift to a later elution volume (consistent with a more positive surface charge), and a new peak began to emerge at ∼14 min. No notable change in total peak area was observed between samples in either chromatographic analysis, implying that there was no loss of protein (i.e., no precipitation) under the test conditions.

**Figure 6:**
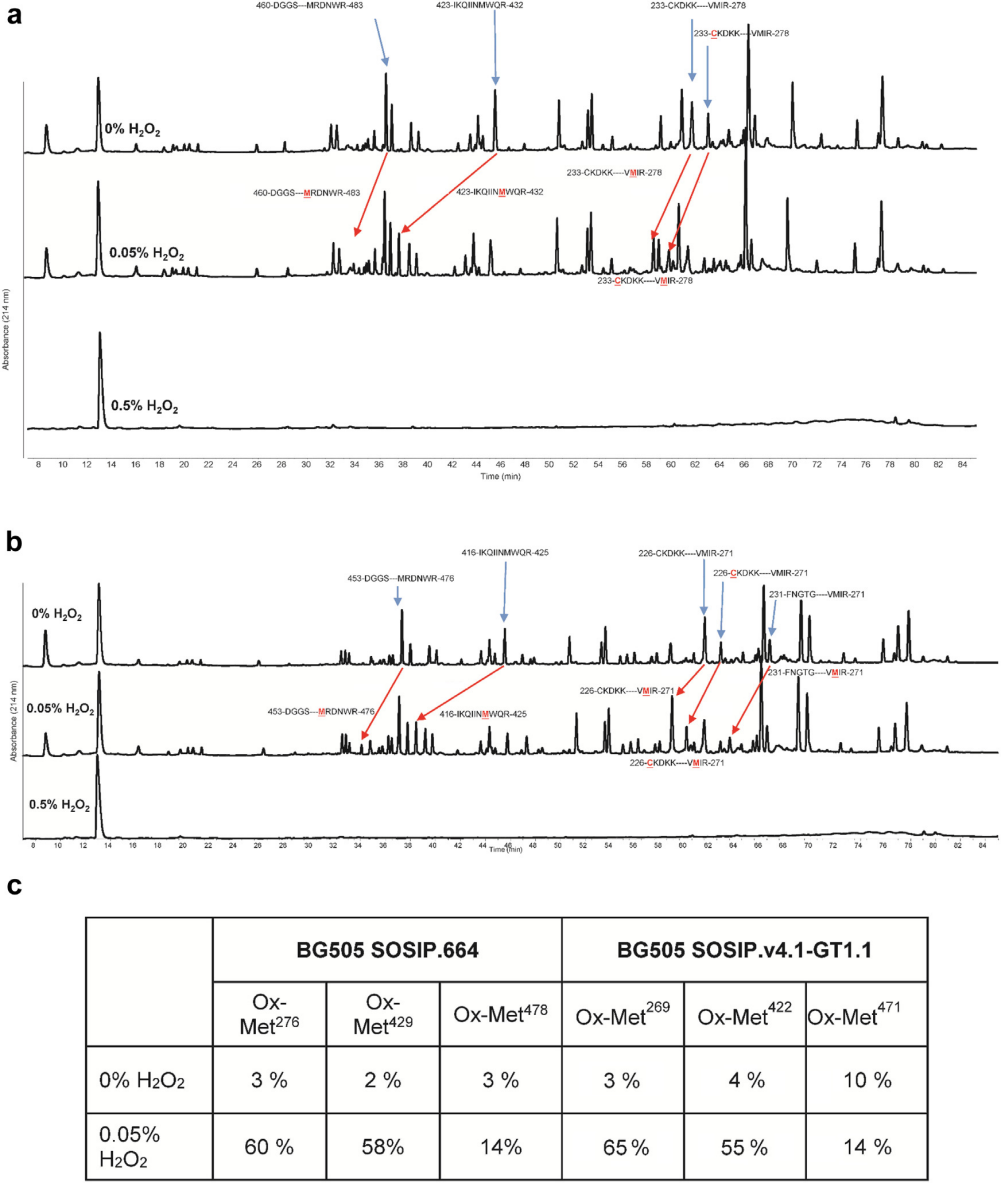
LC-MS peptide map analysis after forced oxidation of SOSIP.664 and GT1.1 gp140 trimers. (a) Representative UV_214nm_ peptide map chromatograms of reduced and trypsin + LysC-digested SOSIP.664 after 1 h incubation with and without 0.5% v/v H_2_O_2_. Samples were evaluated in 10 mM sodium phosphate, 150 mM NaCl (pH 9.0) at room temperature. (b) Representative UV_214nm_ peptide map chromatograms of reduced and trypsin + LysC-digested GT1.1 after 1 h incubation with 0%, 0.05%, or 0.5% v/v H_2_O_2_. Samples were evaluated in 10 mM sodium phosphate, 150 mM NaCl (pH 9.0) at room temperature. The arrows indicate the location of peaks where residues (in red underlined text) are oxidized. (c) Quantitation of oxidized Met residues in BG505 SOSIP.664 and GT1.1 samples incubated with increasing concentrations of H_2_O_2_, as determined by LC-MS peptide mapping. Samples incubated with 0.5% H_2_O_2_ were also evaluated but could not be quantified as no peptides were observed.

Residues susceptible to post-translational modifications under oxidative stress were identified by LC-MS peptide mapping. In total, 80% of the SOSIP.664 or GT1.1 primary sequences were covered in the 0% and 0.05% H_2_O_2_-treated samples after trypsin + LysC proteolysis. This coverage includes 10 of the 12 Met residues present in both proteins (Met^535^ and Met^540^ were not identified). No peptides were observed after proteolysis after exposure of either protein to 0.5% H_2_O_2_ (Fig. 6), which is consistent with the proteins becoming insoluble under these stressed conditions. The relative amounts of post-translational modifications induced by the oxidative stress in each sample were quantified (Fig. 6c). Met oxidation (+16 Da) was identified at the same 3 residues in the SOSIP.664 (Met^271^, Met^426^, and Met^475^) and GT1.1 (Met^271^, Met^426^, and Met^475^) proteins. For SOSIP.664, Met^271^ and Met^426^ were more prone to oxidation than Met^475^ under these conditions, a pattern also seen for GT1.1 (Met^271^ and Met^426^ were more susceptible than Met^475^). A dehydration reaction (indicated by a loss of 18 Da) was also detected in the SOSIP.664 Cys^228^-Arg^273^ and GT1.1 Cys^228^-Arg^273^ peptides. It is likely that this event arose during or after proteolysis, but this supposition requires experimental confirmation. The amino acid numbers, aforementioned, is based on HIV-1 HxB2 numbering system (Supplemental Fig. S1).

### Chemical Stability Profiles of BG505 SOSIP.664 and GT1.1: Elevated Temperature and pH Stresses

**Figure 7:**
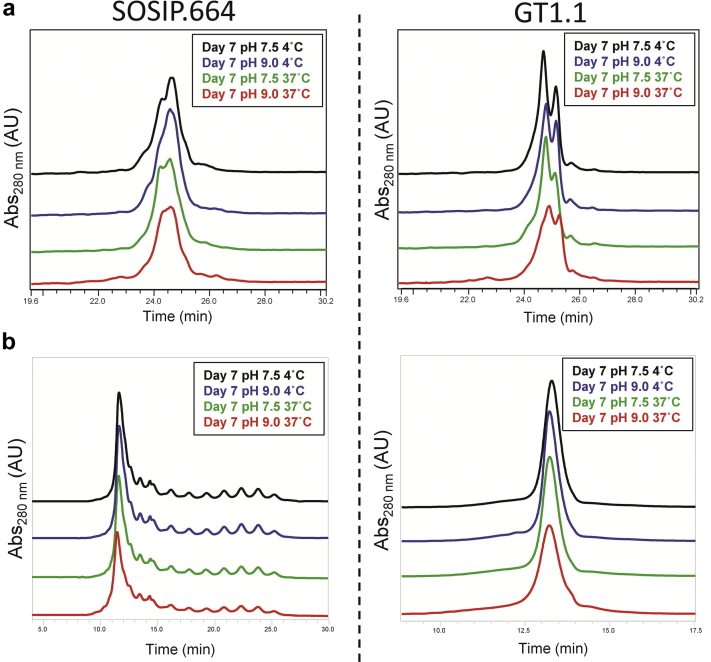
Effect of elevated temperature and pH incubation conditions on BG505 SOSIP.664 and GT1.1 gp140 trimers as measured by 2 chromatographic methods. (a) Representative RP-HPLC analysis of stressed SOSIP.664 and GT1.1 samples. (b) Representative CEX-HPLC analysis of stressed SOSIP.664 and GT1.1 samples. Samples were stored for 7 days at pH 7.5 or 9.0 and at 4°C or 37°C. See Figure 3 for chromatographic profile of unstressed (control) BG505 SOSIP.664 and GT1.1 samples.

**Figure 8:**
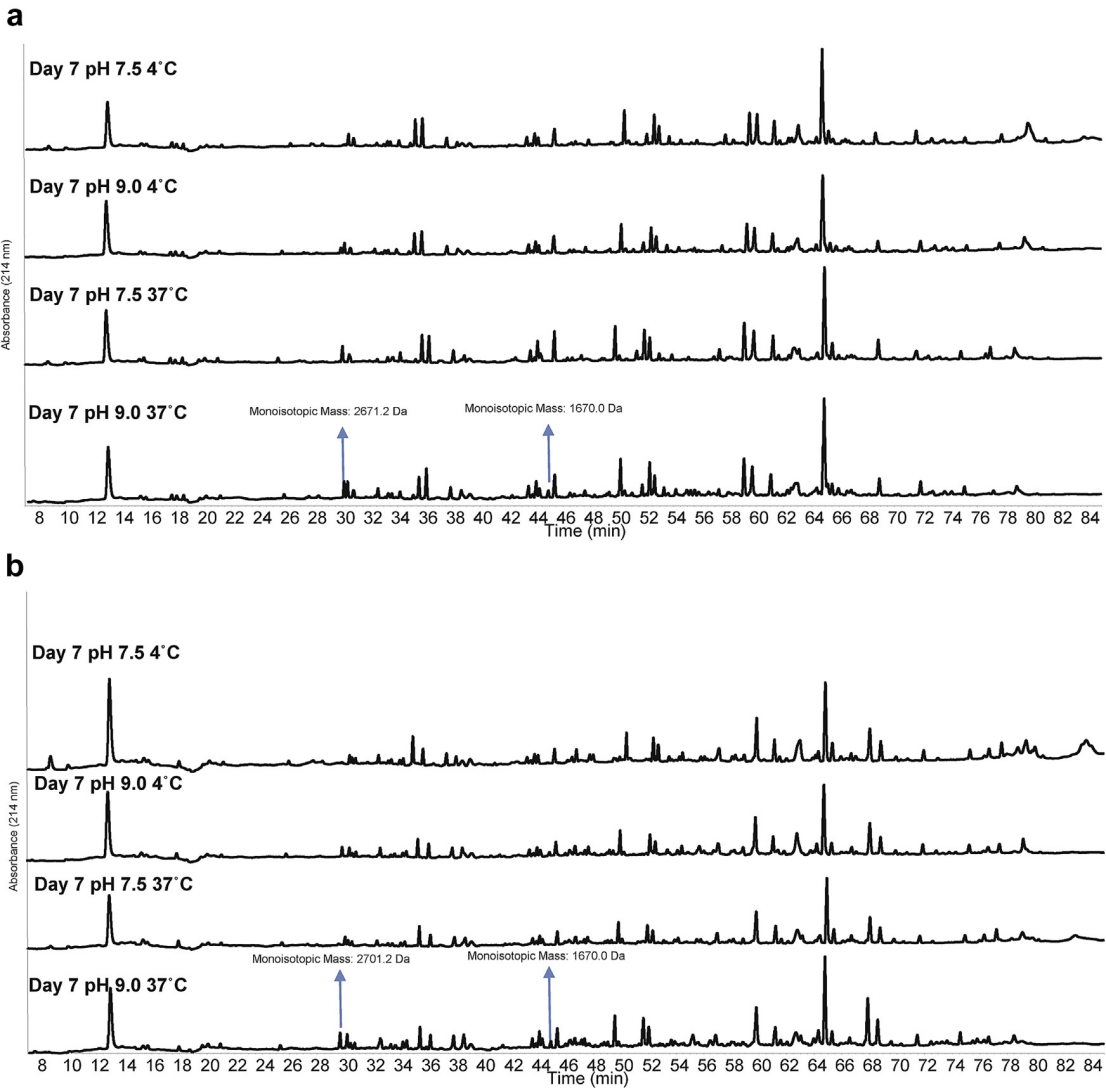
LC-MS peptide map analysis of BG505 SOSIP.664 and GT1.1 gp140 trimers after incubation at different temperature and pH values. (a) Representative UV_214nm_ peptide map chromatograms of reduced and trypsin + LysC-digested SOSIP.664 after 7 days of incubation at 4°C or 37°C at 2 different pH values (7.5 or 9.0). Blue arrows indicate new peaks observed. (b) Same conditions as panel A for GT1.1 sample.

We evaluated the chemical stability of the SOSIP.664 and GT1.1 gp140 trimers by increasing the temperature or pH. Under such conditions, Asn residues can undergo a deamidation reaction through a succinimide intermediate, which results in the formation of iso-aspartate and aspartate residues in the protein.^20^ The SOSIP.664 and GT1.1 gp140 trimers were incubated for 1 week at pH values of 7.5 or 9.0 and at temperatures of 4°C or 37°C, before analysis by RP-HPLC, CEX-HPLC, and LC-MS peptide mapping. In both chromatographic methods, no differences in the elution profiles of either protein were observed between the pH 7.5, 4°C (control) samples and the pH 9.0, 37°C samples (Fig. 7). However, for both trimers, the total peak areas of the pH 9.0, 37°C samples were ∼10% lower compared to the 3 other conditions. This outcome was consistent with protein loss during the 7-day incubation via aggregation and precipitation. We noted that any such precipitated proteins would probably be removed when the samples were centrifuged before RP-HPLC analysis. The peptide chromatograms derived from LC-MS analysis were similar for both trimers under all the test conditions (Fig. 8). Taken together, these results indicated that neither the SOSIP.664 nor GT1.1 gp140 trimer was highly susceptible to Asn deamination under the test conditions of increased pH and temperature.

### Stability of BG505 SOSIP.664 and GT1.1 gp140 Trimers in Common Formulation Buffer: Agitation and Freeze Thaw Stress

An initial formulation of these vaccine antigen candidates (to support Phase I clinical trials) needs to provide sufficient stability for long-term storage as a frozen liquid drug product, followed by thawing and immediate (same day) administration to patients. To this end, we examined if the 2 molecules had similar physical properties on freeze-thaw, not only by using the stress test methods described previously but also by comparing the results with 2 already established stability-indicating test methods (immunoassay and NS-EM analysis).^2^, ^5^ The experiments were performed with the standard formulation buffer (20 mM Tris, 100 mM NaCl, pH 7.5) already used for SOSIP.664^5^, to identify whether any formulation changes were needed for GT1.1 to be stored as a frozen liquid drug product.

**Figure 9:**
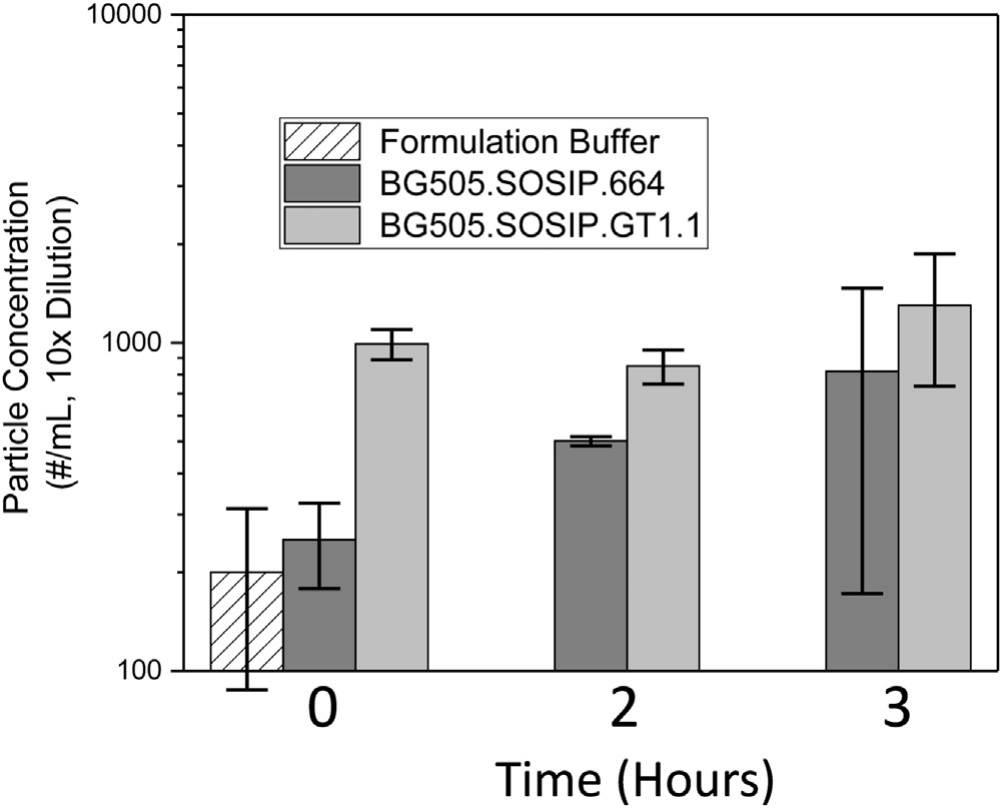
Subvisible (2-100 μm) particle formation (# of particle/mL) during agitation stress studies of BG505 SOSIP.664 and GT1.1 gp140 trimers as determined by MFI. Samples were diluted 10× before analysis and presented without correction. Error bars represent standard deviation from triplicate measurements. Formulation buffer refers to 20 mM Tris, 100 mM NaCl, pH 7.5.

First, the 2 gp140 trimers were subjected to agitation stress to compare their colloidal stabilities in the formulation buffer. The formation of subvisible particles (2-100 μm) was measured by MFI. To conserve the more limited stocks of GT1.1 gp140 trimers during method development, preliminary agitation studies were performed using SOSIP.664, by shaking the samples for up to 72 h at room temperature. The concentration of subvisible particles increased until the 6 h time point, after which a decrease was seen, possibly because larger aggregates formed and settled out. Accordingly, the GT1.1 and SOSIP.664 gp140 trimers were then subjected to agitation stress for up to 6 h (Fig. 9). Although the initial GT1.1 subvisible particle concentration in the formulation buffer was higher than for SOSIP.664, there was no notable increase in the subvisible particle concentration after agitation stress. Thus, the subvisible particle size distributions were similar for both molecules, with most of the particles in the 2-5 μm size range (data not shown).

**Figure 10:**
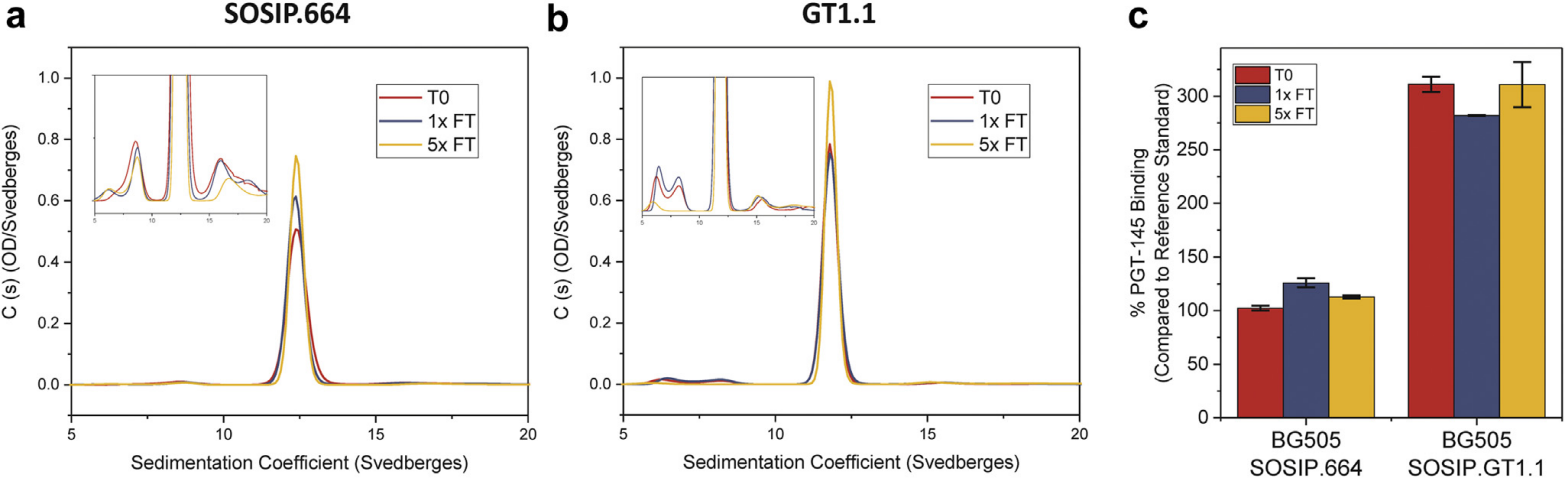
Effect of freeze-thaw stress on stability profile of BG505 SOSIP.664 and GT1.1 gp140 trimers as determined by (a) molecular size distribution as measured by SV-AUC. SV-AUC plots are representative traces from duplicate measurements with *y*-axis zoom shown in inset. (b) PGT145 antibody binding as measured by biolayer interferometry (BLI). Error bars in panel (c) represent standard deviation from triplicate measurements.

Next, the 2 gp140 trimers, in formulation buffer, were frozen at −80°C and thawed at room temperature for 0, 1, and 5 cycles, with their physical stability profiles monitored by SV-AUC and UV-visible spectroscopy. No notable changes in hydrodynamic size were observed for either protein by SV-AUC, as measured by the distribution of sedimentation coefficient values (Fig. 10a). There were also no changes in protein concentration after the freeze-thaw stress, as assayed by UV-visible spectroscopy after centrifugation (data not shown). Methods already established for assaying SOSIP.664 trimer conformational stability included a BLI-based immunoassay using the trimer-specific antibody PGT145 and NS-EM.^2^, ^5^ No notable changes in PGT145 binding were observed before and after subjecting the SOSIP.664 or GT1.1 gp140 trimers to freeze-thaw stress, indicating that the native-like trimer contents remained stable (Fig. 10b). NS-EM imaging before (i.e., no freeze-thaws) and after 3 cycles of freeze-thaw stress (Supplemental Fig. S9) confirmed the SV-AUC and BLI findings. Thus, no non-native Env forms were observed for either vaccine antigen.

## Discussion

A detailed understanding of the structural integrity, physicochemical properties, and stability profiles (under both accelerated and real-time storage conditions) is necessary for the successful formulation development and production of stable clinical dosage forms of any recombinant protein antigen-based vaccine candidate. In the present study, a series of biochemical and biophysical techniques were used in a developability study to compare the BG505 SOSIP.664 gp140 protein antigen to its re-engineered, gI-targeting variant, BG505 SOSIP.v4.1-GT1.1 gp140. The stable nature of this SOSIP.664 gp140 trimer has been determined previously,^5^ but no such studies have been performed on GT1.1. Using SOSIP.664 gp140 trimer as the comparator, we demonstrated that the GT1.1 trimer has similar biophysical properties and pharmaceutical stability profiles under various environmental stresses (e.g., pH, temperature, agitation, and oxidation).

The GT1.1 gp140 trimer differs from SOSIP.664 by 18 amino acid substitutions (resulting in the removal of 5 potential N-glycan sites) and a 7 amino acid deletion in the V2 loop. The 2 trimers had similar tertiary and secondary structures, as judged by second-derivative UV spectroscopy and FTIR spectroscopy (Fig. 1). Small differences in the UV spectroscopy data can possibly be attributed to mutations that result in the addition or removal of aromatic residues (i.e., A316W and Y173H). However, the hydrophobicities and surface charges of the 2 trimers did differ (Figs. 3a and 3b). Particularly striking is the presence of ∼10 minor peaks only in the SOSIP.664 CEX-HPLC chromatograms (Fig. 3b). Differences in the characteristics of the extensive array of N-linked glycans present on the 2 trimers might underpin this finding.^19^ More specifically, the much simpler elution profile of the GT1.1 trimer might reflect the absence of 5 potential N-glycan sites compared to its SOSIP.664 counterpart. Additional analyses would, however, be needed to identity the minor peaks, such as by fraction collection followed by LC-MS. These striking chromatographic differences were not apparent in the RP-HPLC data, which only show minor differences in the elution profiles. An H_2_O_2_ stress test analysis suggests that the latter differences are oxidation-dependent (Fig. 5a).

Similar physical stability profiles of the SOSIP.664 and GT1.1 gp140 trimers were noted by comparisons of their temperature and pH behavior as measured by 3 different biophysical techniques and assessed by data visualization tools. The 3-index EPD stability profiles generated from the biophysical stability data sets are similar for both trimers (Fig. 4), with no notable differences in the defined structural regions. The only methods showing notable differences in the structures/post-translational modifications of the 2 protein molecules were RP-HPLC and CEX-HPLC. Even so, both proteins were similarly susceptible to oxidation and deamidation reactions under stressed conditions (Figure 5, Figure 6, Figure 7, Figure 8). Thus, any differences in the prestress (i.e., control) samples do not translate into variations in whether and how chemical changes occur during accelerated stability testing. The similar outcomes of the physicochemical stability tests can be indicative of comparable storage stability profiles for the 2 SOSIP gp140 trimers under real-time conditions.^21^ We suggest, therefore, that the BG505 SOSIP.v4.1-GT1.1 gp140 trimer is likely to be as stable as the prototypic SOSIP.664 gp140 trimer, while noting that long-term stability studies will be required to confirm this supposition.

The formulation, storage, and administration of vaccine antigens for an initial phase 1 clinical trial is typically designed based on stricter, more well-controlled conditions (e.g., administration by a medical professional at 1 clinical site), which would not be practical for wide distribution of commercial formulations. For example, vaccine antigens can be stored frozen as bulk drug substances to ensure long-term stability, and then thawed, formulated for fill as drug products before they are mixed with adjuvants (“bedside” mix) and immediately administered to a patient. To this end, the aggregation propensities and overall stabilities of the GT1.1 and SOSIP.664 gp140 trimers were assessed during agitation and freeze-thaw and found to be comparable when both trimers were formulated in the same simple buffer (20 mM Tris, 100 mM NaCl, pH 7.5) (Figure 9, Figure 10, and Supplemental Fig. S9). This outcome was observed using the orthogonal methods of SV-AUC and BLI immunoassay and confirmed by NS-EM imaging, a technique previously utilized to evaluate trimer stability after freeze-thaw cycles.^5^, ^22^

The freeze-thaw stability results are somewhat surprising given the formulation contains 2 excipients (Tris buffer and NaCl) with known incompatibilities with proteins during freeze-thaw stress.^23^ First, the pH of Tris-buffered solutions is temperature-dependent,^24^ which can lead to protein instability because of pH changes during freeze-thaw cycles. Thus, when a Tris buffer is adjusted to pH 7.5 at 25°C, its pH will increase to ∼8.1 at 5°C and to ∼8.25 at 0°C.^25^ Despite this factor, we saw no instability on freeze-thawing, which indicates that both trimers remain physically stable under these pH conditions. Concerns over the aforementioned temperature-dependent pH shifts prompted preliminary stability studies to be performed using Histidine and HEPES buffer systems, but no notable changes in stability (and hence no improvements) were observed in these tests (data not shown). Furthermore, the resilience of SOSIP.664 gp140 to alkaline pHs has been observed previously.^5^ A second factor is that sodium chloride is known to concentrate during freezing, which can increase the ionic strength of solutions.^25^ But again, as we saw no physical instability in the freeze-thaw studies, we can infer that both trimers remain physically stable under these ionic strength conditions. We did perform preliminary stability studies using 10% sucrose instead of NaCl as a cryoprotectant and tonicifying agent, but again found no notable changes in stability (and hence no improvements).

In summary, engineering the native-like GT1.1 gp140 trimers to bind gl-bNAb precursors is a plausible approach to initiating the induction of bNAbs as a HIV-1 vaccine strategy.^1^ However, modifications to the protein sequence (that can also lead to differences in glycosylation profile) may impair the conformational, chemical, and colloidal stability properties of a vaccine antigen. Each successive iteration of the prototypic BG505 SOSIP.664 gp140 trimer must therefore be evaluated by methodologies similar or equivalent to those described here, to assess the pharmaceutical stability of the new antigen and identify whether additional formulation development work is required for initial clinical development using a frozen liquid formulation. Here, we characterized both the BG505 SOSIP.664 gp140 trimer prototype and its new GT1.1 variant, using a battery of physicochemical methods and compared their physicochemical stability profiles versus pH, temperature, agitation, and freeze-thaw stresses. We conclude from this developability assessment that the BG505 SOSIP.v4.1-GT1.1 gp140 vaccine candidate is similar in its overall structural stability to the SOSIP.664 gp140 trimer, from which it was engineered, from a formulation development point of view. Hence, similar formulation conditions and processes can be used for the existing (SOSIP.664) and new (GT1.1) vaccine drug product candidates.^5^
