# Supplementary material for: Developability Assessment of Physicochemical Properties and Stability Profiles of HIV-1 BG505 SOSIP.664 and BG505 SOSIP.v4.1-GT1.1 gp140 Envelope Glycoprotein Trimers as Candidate Vaccine Antigens
Source: J Pharm Sci. 2019 Jul;108(7):2264–77. doi: 10.1016/j.xphs.2019.01.033 (PMC6595180; doi:10.1016/j.xphs.2019.01.033)
Supplement: Supplemental Information [file mmc1.docx]

**Supplemental Material:**

**Developability assessment of physicochemical properties and stability profiles of HIV-1 BG505 SOSIP.664 and BG505 SOSIP.v4.1-GT1.1 gp140 envelope glycoprotein trimers as candidate vaccine antigens**

*Neal Whitaker^1^, John M. Hickey^1^, Kawaljit Kaur^1^, Jian Xiong^1^, Nishant Sawant^1^, Albert Cupo^2^, Wen-Hsin Lee^3^, Gabriel Ozorowski^3^, Max Medina-Ramírez^4^, Andrew B. Ward^3^, Rogier W. Sanders^2,4^, John P. Moore^2^, Sangeeta B. Joshi^1^, David B. Volkin^1*^, and Antu K. Dey^5*^*

*^1^Department of Pharmaceutical Chemistry, Macromolecule and Vaccine Stabilization Center, University of Kansas, Lawrence, KS, 66047, USA.*

*^2^Department of Microbiology and Immunology, Weill Medical College of Cornell University, New York, NY 10065, USA.*

*^3^Department of Integrative Structural and Computational Biology, Center for HIV/AIDS Vaccine Immunology and Immunogen Discovery, International AIDS Vaccine Initiative Neutralizing Antibody Center, and Collaboration for AIDS Vaccine Discovery, The Scripps Research Institute, La Jolla, California, USA.*

*^4^Department of Medical Microbiology, Academic Medical Center, University of Amsterdam, Amsterdam, The Netherlands.*

*^5^International AIDS Vaccine Initiative, New York, NY 10004 USA.*


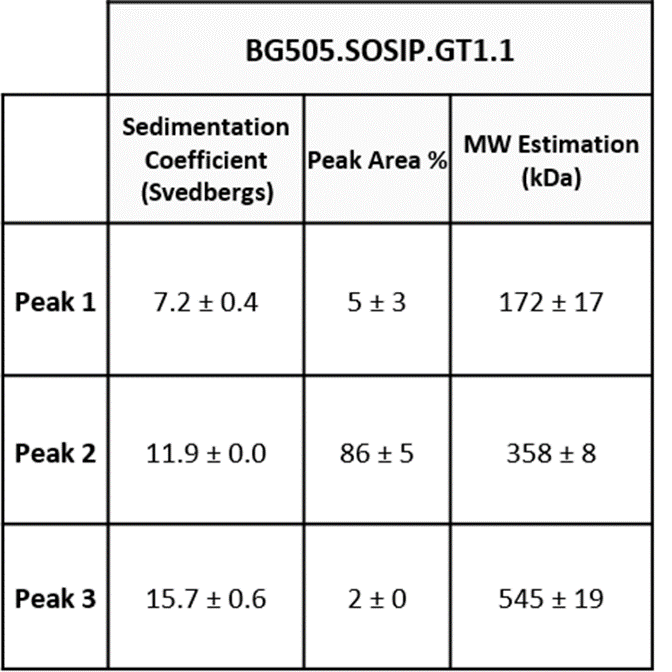

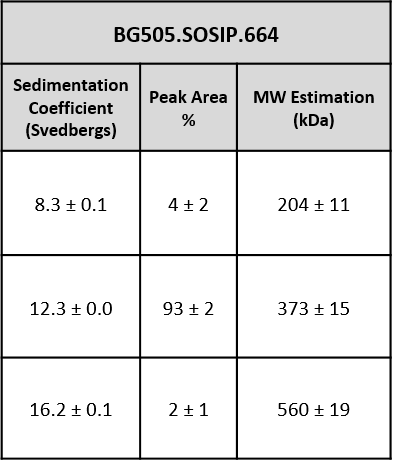


**Supplementary Table S1:** Sedimentation coefficient distribution values, percent area and molecular weight (MW) estimates (in KDa) of BG505 SOSIP.664 and GT1.1 samples as measured by SV-AUC. Mean ± SD (standard deviation) from triplicate measurements are reported.


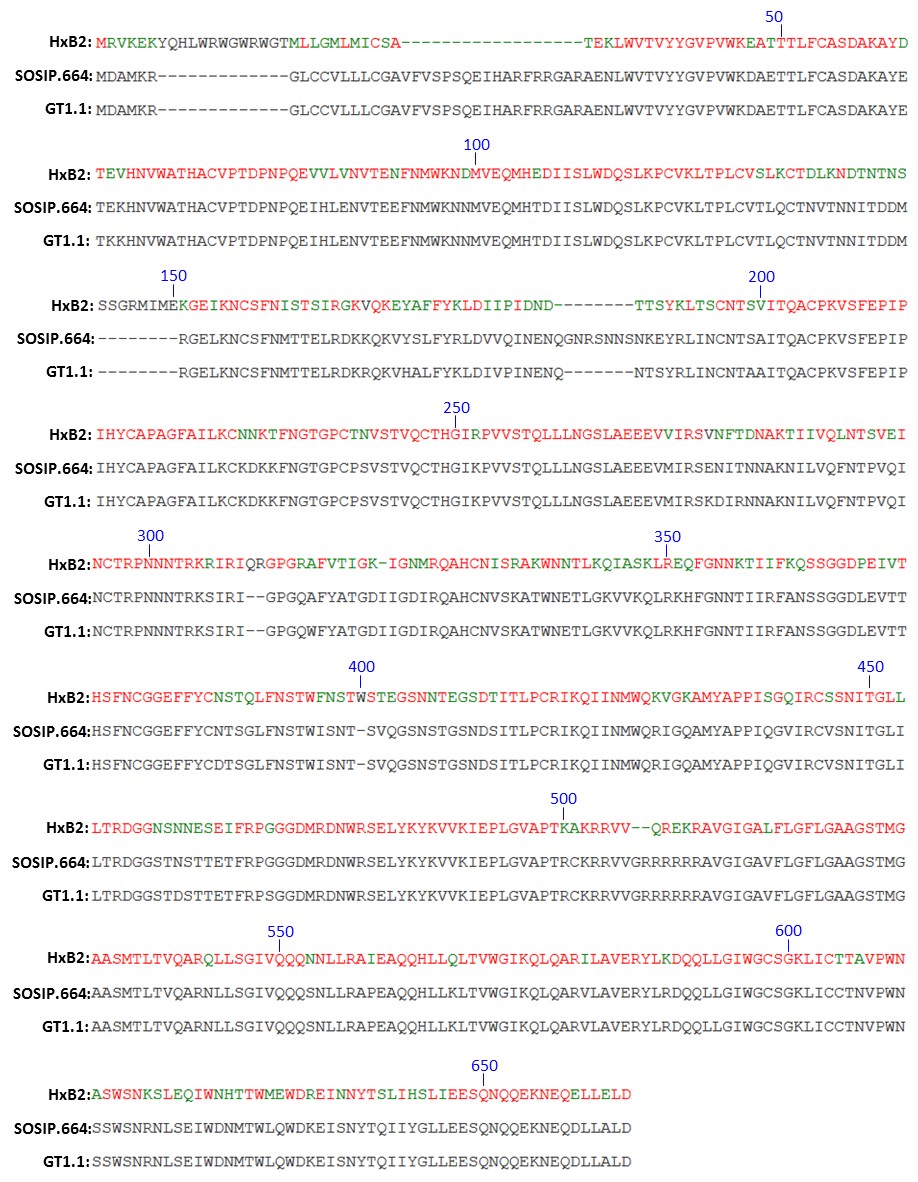


**Supplemental Figure S1:** Amino acid sequence alignment of BG505 SOSIP.664 (SOSIP.664) and BG505 SOSIP.v4.1-GT1.1 (GT1.1) gp140. HxB2 is used as reference to cite the amino acid numbering because it is a standard to number positions in HIV sequence relative to HxB2. The color coding in HxB2 is: red - 100% Identity, blue - 99-75% Identity, and green - 74%-50% Identity.

**
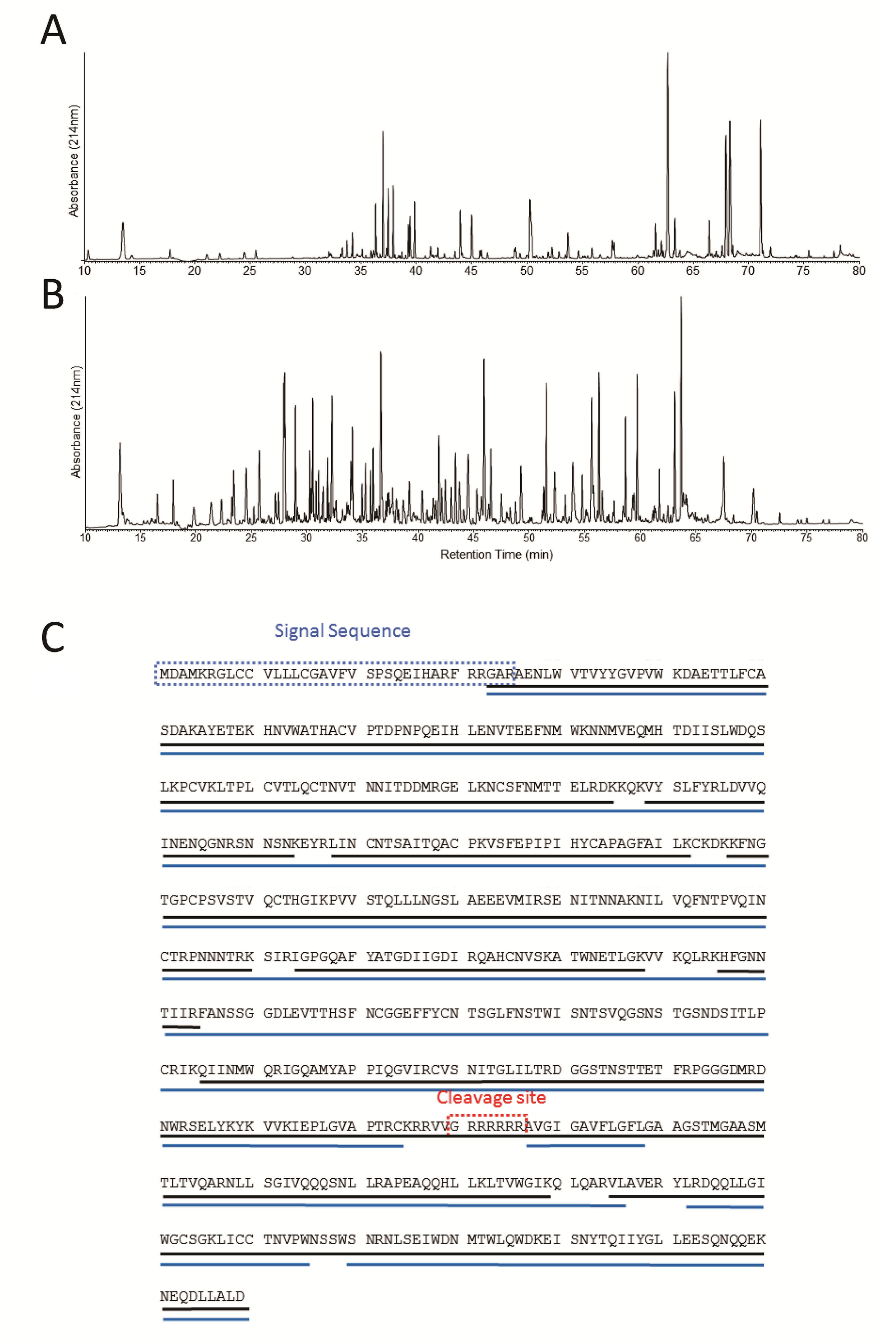
Supplemental Figure S2:** LC-MS peptide mapping of BG505 SOSIP.664. Representative UV214nm chromatograms of reduced and A) Trypsin + LysC or B) Chymotrypsin digested SOSIP.664. C) Primary sequence coverage of SOSIP.664 digested using either Trypsin + LysC (black bars) or Chymotrypsin (blue bars). Residues comprising the signal sequence and cleavage site are boxed in blue and red, respectively. Note that overlapping peptides are not depicted in this figure.


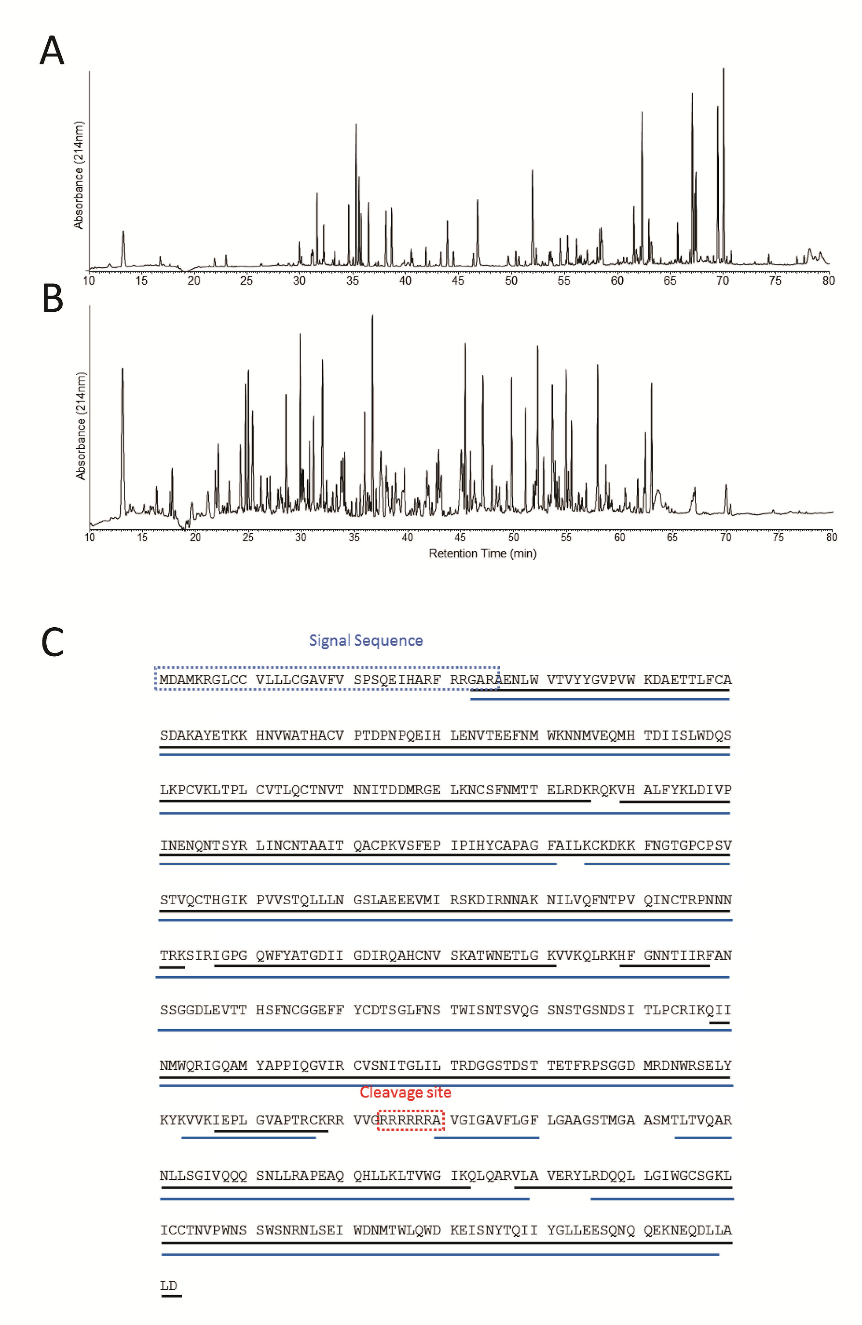


**Supplemental Figure S3:** LC-MS peptide mapping of BG505 SOSIP.v4.1-GT1.1. Representative UV214nm chromatograms of reduced and A) Trypsin + LysC or B) Chymotrypsin digested GT1.1. C) Primary sequence coverage of GT1.1 digested using either Trypsin + LysC (black bars) or Chymotrypsin (blue bars). Residues comprising the signal sequence and cleavage site are boxed in blue and red, respectively. Note that overlapping peptides are not depicted in this figure.


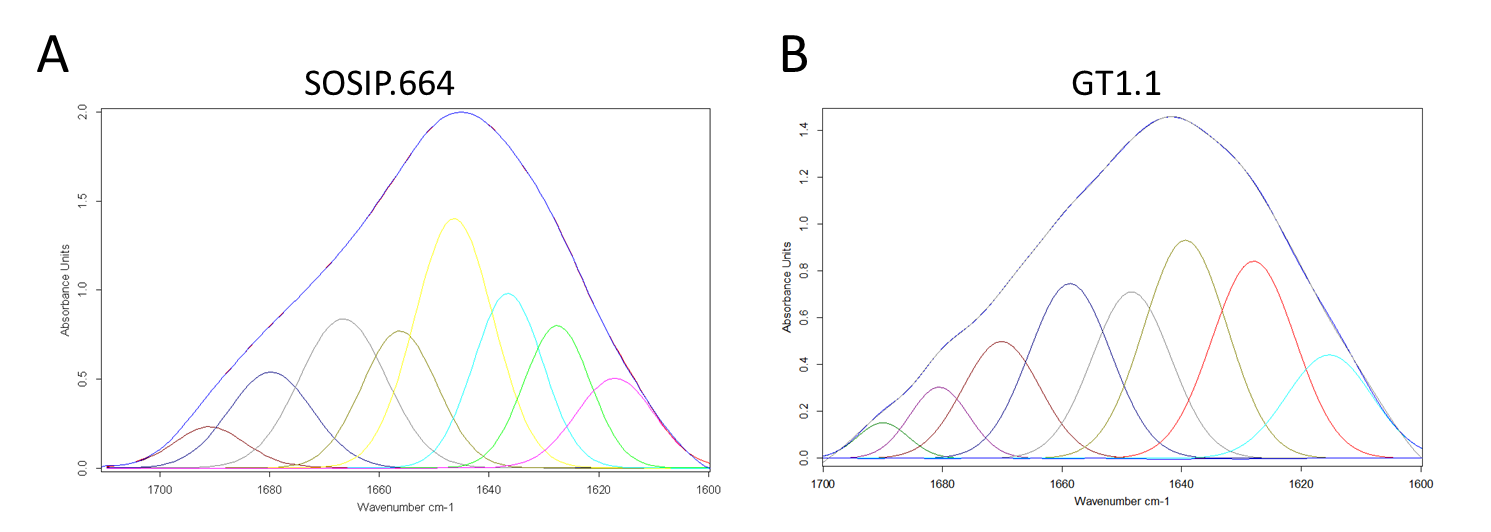


**Supplemental Figure S4**: FTIR spectra of SOSIP.664 (A) and GT1.1 (B) as fitted to determine secondary structure content. Following Fourier self-deconvolution, 8 peaks were fitted to the IR spectrum in the Amide I region (1700-1600 cm-1) using a mixed Gaussian and Lorentzian function. The areas of the peaks were used to determine the relative percentage of secondary structure components in the trimer samples.


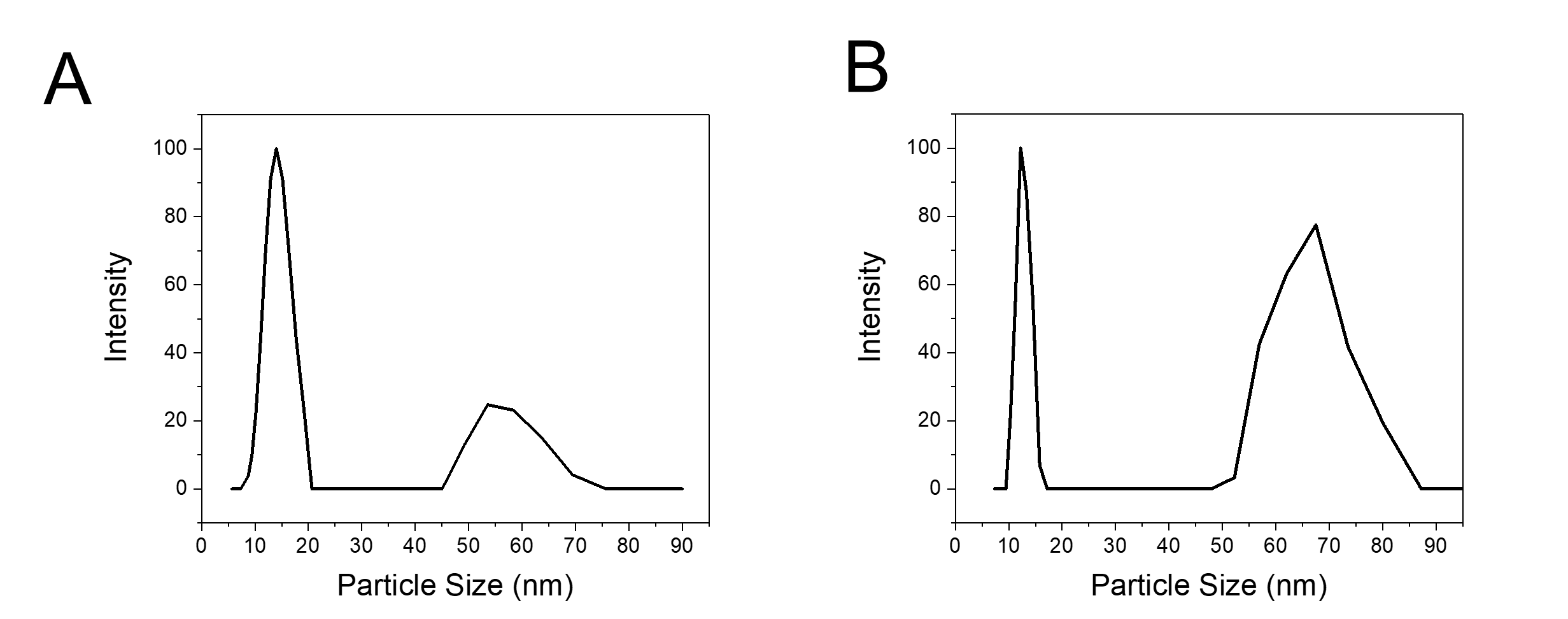


**Supplemental Figure S5:** DLS analysis of BG505 SOSIP.664 and GT1.1 trimers. Intensity weighted MSD analyses of A) SOSIP.664 and B) GT1.1 are shown.


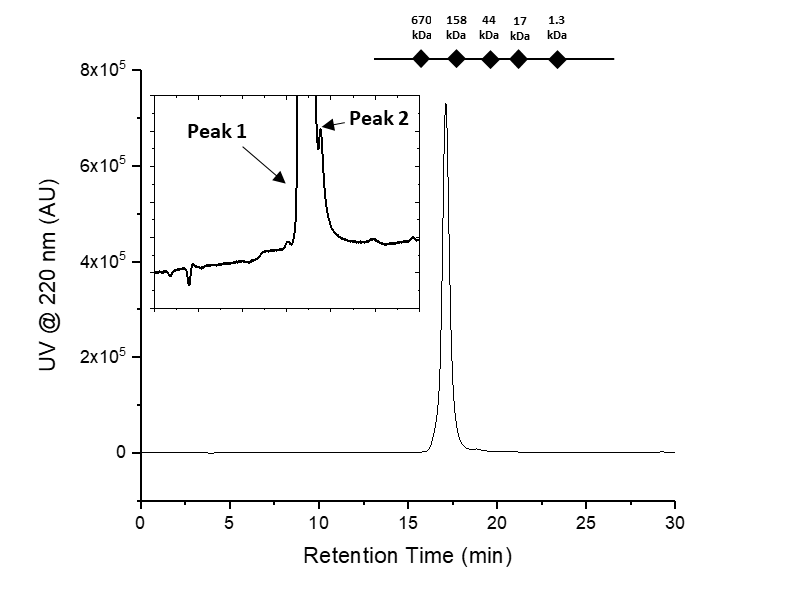


**Supplemental Figure S6:** Representative SE-HPLC analysis of SOSIP.664. The inset shows zoomed section of the peak area where a minor peak (peak 2) is seen in addition to the main peak (peak 1). Molecular weight (MW) standard positions are indicated, above the chromatogram, with black diamonds with the MW (in KDa) indicated.


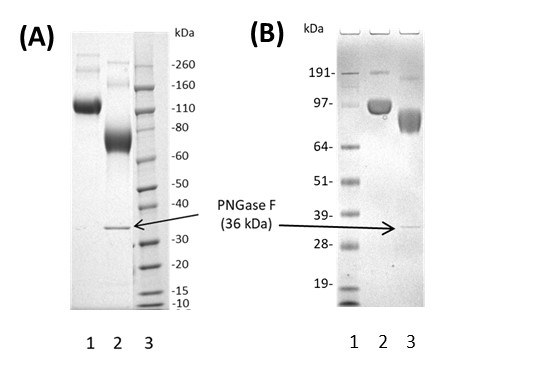


**Supplemental Figure S7:** SDS-PAGE analysis of BG505 SOSIP.664 and GT1.1 gp140 trimers after treatment with PNGase F. (A) SOSIP.664 - Lane 1 contains (control/undigested) SOSIP.664 gp140, lane 2 contains PNGase F digested SOSIP.664 gp140, and lane 3 contains molecular weight standards. (B) GT1.1 – Lane 1 contains molecular weight standards, lane 2 contains (control/undigested) GT1.1 gp140, and lane 3 contains PNGase F digested GT1.1 gp140. PNGase F bands in both gels (in PNGase F treated sample lanes) are pointed by arrows. Both gels are visualized using Coomassie staining.


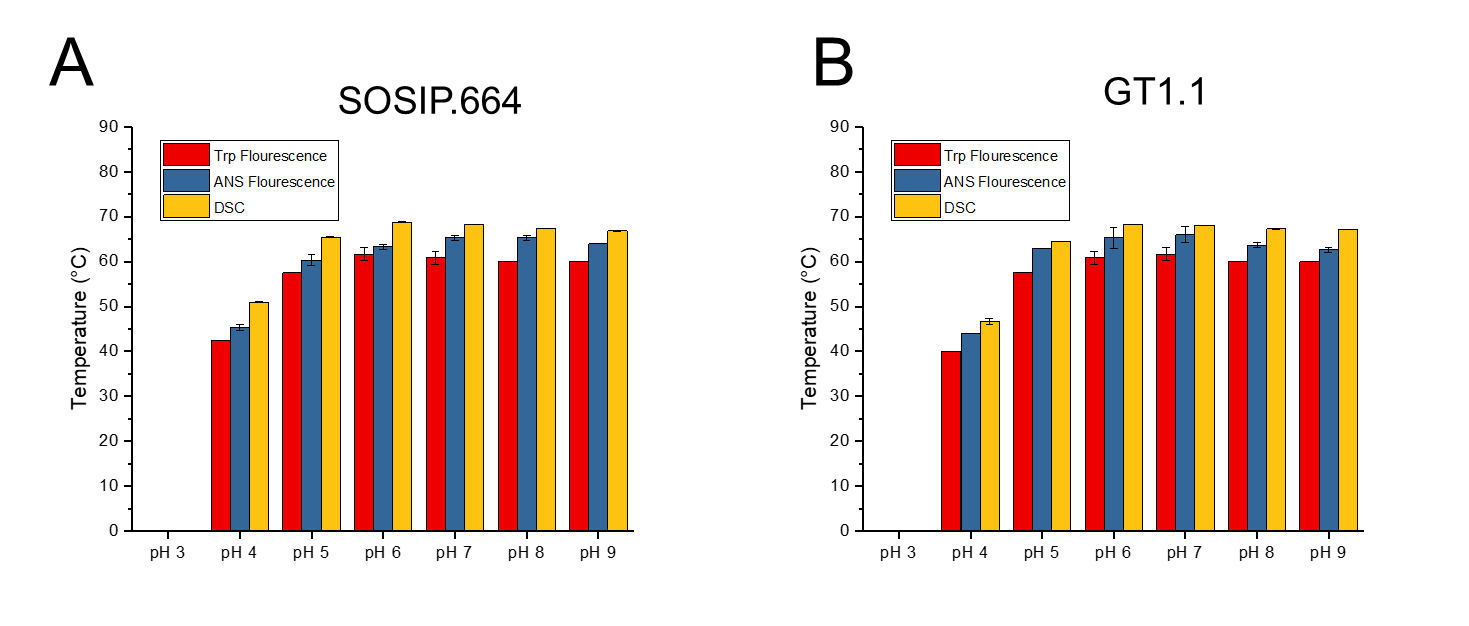


**Supplemental Figure S8:** Thermal melting temperatures (Tm) values for BG505 SOSIP.664 (A) and GT1.1 (B) trimers from intrinsic tryptophan fluorescence (red), extrinsic ANS fluorescence (blue) and DSC (yellow) as a function of solution pH (in a citrate-phosphate buffer containing sodium chloride). Error bars represent standard deviation from triplicate measurements.


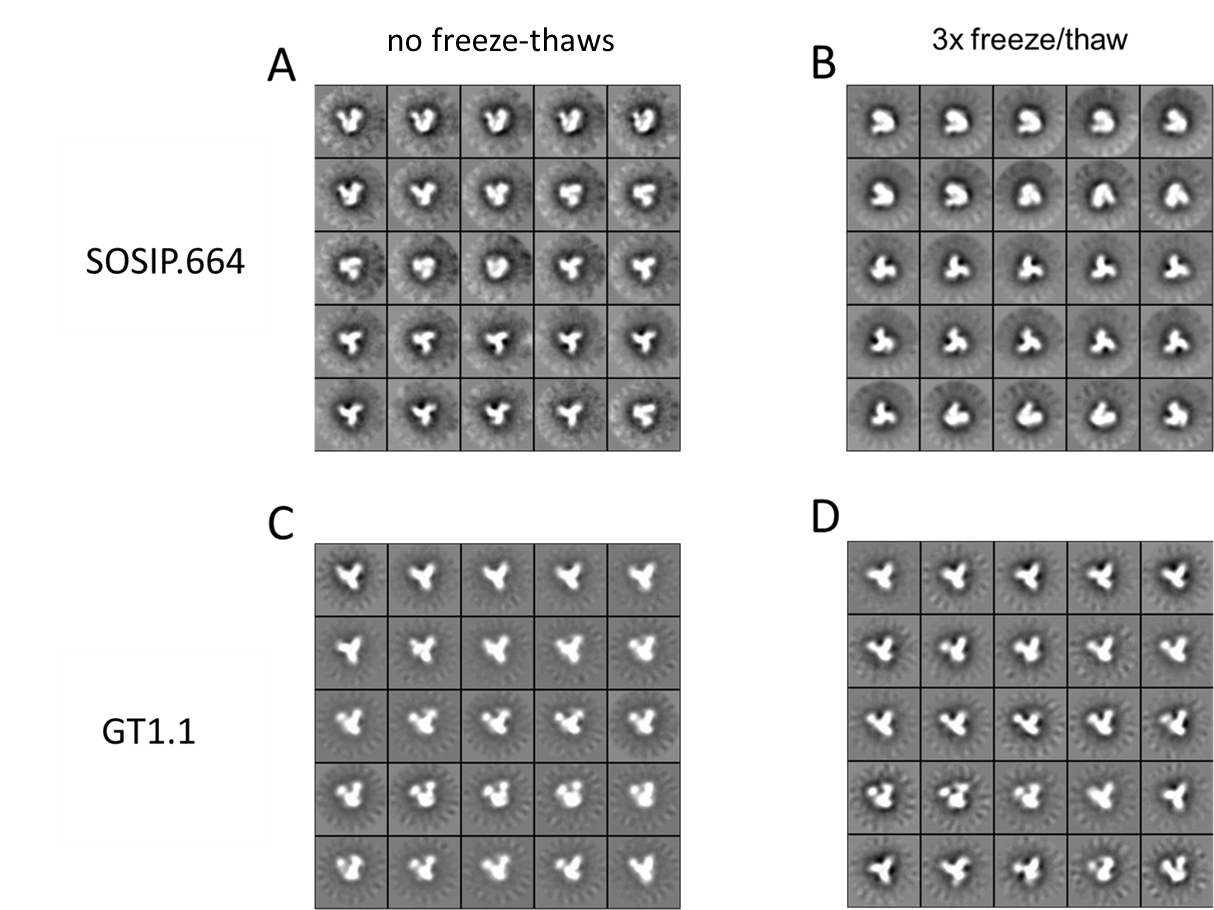


**Supplemental Figure S9:** 2D class averages of NS-EM analyses of (A, B) BG505 SOSIP.664 trimers and (C, D) GT1.1 trimers after the indicated number of freeze-thaw cycles.
